# Comparative study of unsupervised dimension reduction techniques for the visualization of microarray gene expression data

**DOI:** 10.1186/1471-2105-11-567

**Published:** 2010-11-18

**Authors:** Christoph Bartenhagen, Hans-Ulrich Klein, Christian Ruckert, Xiaoyi Jiang, Martin Dugas

**Affiliations:** 1 Department of Medical Informatics and Biomathematics, University of Münster, Domagkstraße 9, 48149 Münster, Germany; 2 Department of Computer Science, University of Münster, Einsteinstrasse 62, 48149 Münster, Germany

## Abstract

**Background:**

Visualization of DNA microarray data in two or three dimensional spaces is an important exploratory analysis step in order to detect quality issues or to generate new hypotheses. Principal Component Analysis (PCA) is a widely used linear method to define the mapping between the high-dimensional data and its low-dimensional representation. During the last decade, many new nonlinear methods for dimension reduction have been proposed, but it is still unclear how well these methods capture the underlying structure of microarray gene expression data. In this study, we assessed the performance of the PCA approach and of six nonlinear dimension reduction methods, namely Kernel PCA, Locally Linear Embedding, Isomap, Diffusion Maps, Laplacian Eigenmaps and Maximum Variance Unfolding, in terms of visualization of microarray data.

**Results:**

A systematic benchmark, consisting of Support Vector Machine classification, cluster validation and noise evaluations was applied to ten microarray and several simulated datasets. Significant differences between PCA and most of the nonlinear methods were observed in two and three dimensional target spaces. With an increasing number of dimensions and an increasing number of differentially expressed genes, all methods showed similar performance. PCA and Diffusion Maps responded less sensitive to noise than the other nonlinear methods.

**Conclusions:**

Locally Linear Embedding and Isomap showed a superior performance on all datasets. In very low-dimensional representations and with few differentially expressed genes, these two methods preserve more of the underlying structure of the data than PCA, and thus are favorable alternatives for the visualization of microarray data.

## Background

DNA microarrays allow the measurement of transcript abundances for thousands of genes in parallel. Applications in quality assessment and interpretation of such high dimensional data by clustering [1,2] and visualization [3,4] make use of algorithms that reduce its dimension. Two and three dimensional visualizations are often a good way to get a first impression of properties or the quality of a dataset or of special patterns within the data by showing clusters such as diseased and healthy patients, revealing outliers, a high level of noise or to generate hypotheses for further experimentation [5-8]. In general, there are two different approaches to reduce a datasets' dimension. Feature selection methods [9-11] compute a ranking on all genes by means of some given score and pick a gene subset based on this ranking. Feature extraction methods define a mapping between the high-dimensional input space and a low-dimensional target space of a given dimension. Both methods are used in machine learning concepts. Most classification algorithms use many or all features in a complex (nonlinear) manner whereas approaches like [12,13] are based on the relative expression of only two or three genes to overcome the "black box" character of the other classifiers. So they allow an easy traceability of the genes leading to the classification result. On the other hand, applications like the visualization of high-dimensional data may profit from extracting information from all features. This results in feature extraction methods usually being more suited for low-dimensional representations of the whole data. In the following, we refer to feature extraction methods when speaking of dimension reduction techniques.

Considering visualization, these kind of mappings are often unsupervised, because they don't use further information of the data like class labels and allow an unbiased view of the structure within the data. Supervised methods are more applicable to improve classification or regression procedures, assuming that less non-differential or noisy features are reduced after the mapping.

All features, that are related to special properties of the data or a separation into classes or clusters, often lie in a subspace of a lower (intrinsic) dimension within the original data. A 'good' dimension reduction technique should preserve most of these features and generate data with similar characteristics like the high-dimensional original. For example, classifications should work at least as well on the low-dimensional representation and clusters within the reduced data should also be found, preferably more distinct. Principal Component Analysis (PCA) is a widely used unsupervised method to define this mapping from high-to low-dimensional space. Availability of large datasets with high-dimensional data, especially in biological research (e.g. microarrays), led to many new approaches in the last years.

Other studies, that deal with the assessment of dimension reduction techniques, either compare them against the background of classification [14-18], and hence mainly discuss supervised methods like Partial Least Squares [19,20], Sliced Inverse Regression [21] or other Regression models [22], or come from Computer Vision and deal with text, image, video or artificial data like the Swiss Roll [23-28]. This study instead, focuses on microarray data and its two and three dimensional visualization. We compare PCA to six recent unsupervised methods to find out if and under which conditions they are able to outperform PCA. In the following sections, we describe a benchmark, consisting of classifications and cluster validations, to compare the visualization performance of seven dimension reduction techniques on ten real microarray and several simulated datasets. After some technical details in the methods section, we present and discuss all results, based on one representative dataset. Further details of the other nine datasets are available in the supplement.

## Methods

### Dimension Reduction

Seven unsupervised dimension reduction techniques were compared within this study: Principal Component Analysis (PCA), Kernel PCA (KPCA), Isomap (IM), Maximum Variance Unfolding (MVU), Diffusion Maps (DM), Locally Linear Embedding (LLE) and Laplacian Eigenmaps (LEM). These dimension reduction techniques can be divided into two groups: linear and nonlinear methods. While PCA belongs to the former, due to a linear combination of the input data, the other six methods were designed with respect to data lying on or near a nonlinear submanifold in the higher dimensional input space and perform a nonlinear mapping.

Given an input space ℝ*^D ^*and target space ℝ*^d ^*(with *d *<<*D*) let *X *∈ ℝ^*N*×*D *^be an input dataset of *N *samples and *D *features (gene expression values) and *Y *∈ ℝ^*N*×*d *^its low-dimensional representation. A dimension reduction technique is a mapping *Φ*: ℝ*^D ^*→ ℝ*^d ^*that optimizes a cost function *∈ *: *R^d ^*→ ℝ on the target space. This problem can often be reduced to an eigenvalue problem, whose eigenvectors will define the embedding *Y *.

### Principal Component Analysis

Principal Component Analysis (PCA) [29,30] builds a new coordinate system by selecting those *d *axes *w*_1_*, . . . , w_d _*∈ ℝ*^D^*, which maximize the variance in the data:

$$w_{1}=\arg\underset{\|w\|=1}{\max}\text{var}\left(Xw\right)=\arg\underset{\|w\|=1}{\max}w^{\prime }Cw,$$

*w*_2_*, . . . , w_d _*are chosen in the same way, but orthogonal (independent) to each other (here, *C *∈ ℝ*^DxD ^*denotes the covariance matrix of the data *X*). So, the principal components *p_i _*= *Xw_i _*explain most of the variance in the data. Before mapping the data, the samples in *X *were centered by subtracting their mean. Since PCA only considers the variance among samples, it works best if those features, that are relevant for class labeling, account for a large part of the variance. Sometimes, the first two or three principal components are not sufficient for a good representation of the data [26]. This can lead to a high target dimensionality and prevent a well suited visualization. Furthermore, the covariance matrix grows rapidly for high-dimensional input data. To overcome this issue, we substituted the covariance matrix by the matrix of squared Euclidean distances $D_{E}=\frac{1}{N}XX^{\prime }\left(D_{E}\in {\mathbb{R}}^{N\times N}\right)$[14,31].

### Kernel PCA

To make PCA more suitable for nonlinear data, Kernel PCA (KPCA) maps the data into a higher dimensional feature space before applying the the same optimization as PCA. [32,33]. The mapping can be done implicitly by using a kernel function. The Gaussian kernel $K\left(x_{i},x_{j}\right)=exp\left(\frac{-{\left\|x_{i}-x_{j}\right\|}^{2}}{\sigma^{2}}\right)$ was applied in our study.

### Isomap

Isomap (IM) [27,28], a nonlinear modification of Multidimensional Scaling [34], preserves the global structure of the input data in its low-dimensional representation. This is done by constructing a neighborhood graph *G*, weighted by shortest geodesic distances *D_G _*∈ ℝ*^N×N ^*between all *k *nearest neighbors. This way, Isomap captures paths along a nonlinear manifold instead of the direct Euclidean distance. The embedding into the low-dimensional space is done by selecting *y*_1_*, . . . , y^d ^*∈ ℝ*^N ^*such that

$$\epsilon ={\left\|D_{G}-D_{Y}\right\|}_{L^{2}}$$

,is minimized, with $D_{Y}\left(i,j\right)={\left\|y_{i}-y_{j}\right\|}^{2}$ being the pairwise distance matrix of neighbors *y_i _, y_j _*in the target space.

Previous work in [23] addressed problems in visualizing datasets consisting of several well separated clusters. Since Isomap is known to suffer from holes in the underlying manifold [14], it is suggested to modify the method by selecting $\frac{k}{2}$ nearest and $\frac{k}{2}$ farthest neighbors when constructing the graph, instead of the *k *nearest neighbors. Both, IM and IM(mod), will be discussed in the results section.

### Maximum Variance Unfolding

Similar to Isomap, Maximum Variance Unfolding (MVU) [25,26] preserves the distances among *k *nearest neighbors by means of a neighborhood graph *G*. But it varies in considering squared Euclidean distances between two neighbored samples, instead of geodesic distances and in maximizing the Euclidean distance between all points *y_i _, y_j _*in the target space (to 'unfold' the data) while preserving the distances in the neighborhood graph. This leads to the optimization problem.

$$max\sum_{ij}{\left\|y_{i}-y_{j}\right\|}^{2}\text{subject to}D_{G}=D_{Y}$$

Based on the same concept, MVU shares some weaknesses with Isomap like suffering from erroneous connections in the graph.

### Diffusion Maps

Diffusion Maps (DM) [35,36] start with building a graph *G *as well, but differ in weighting the edges by the Gaussian kernel function: $W\left(i,j\right)=\exp\left(\frac{-{\left\|x_{i}-x_{j}\right\|}^{2}}{\sigma^{2}}\right)$. With the rows being normalized by $\hat{W}\left(i,j\right)=\frac{W\left(i,j\right)}{\Sigma_{l=1}^{N}W\left(i,l\right)}$, the weights $\hat{W}\in {\mathbb{R}}^{N\times N}$ can be seen as a Markov Matrix that defines the probability to move from one sample to another in one time step. The transition probability for *t *time steps, denoted ${\hat{W}}^{\left(t\right)}$, is given by ${\hat{W}}^{t}$. It can be used to control the local connections among neighbored samples. Here, we set it to *t *= 1. Diffusion Maps retain a weighted *L*^2 ^distance, the 'diffusion distance'

$$D^{\left(t\right)}\left(x_{i},x_{j}\right)=\sqrt{\sum_{l=1}^{N}\frac{{\left({\hat{W}}^{\left(t\right)}\left(i,l\right)-{\hat{W}}^{\left(t\right)}\left(j,l\right)\right)}^{2}}{\psi \left(xl\right)}}$$

The term $\Psi \left(x_{i}\right)=\frac{\Sigma_{j}\hat{W}\left(i,j\right)}{\Sigma_{jl}\hat{W}\left(j,l\right)}$ leads to stronger weighting of samples from dense areas in the graph. Since the diffusion distance between two points is computed over all possible paths in the graph, Diffusion Maps are more robust to noise.

### Locally Linear Embedding

Unlike Isomap and MVU, Locally Linear Embedding (LLE) [24,37] attempts to preserve local properties of the data. Each sample *x_i _*is represented by a linear combination of its *k *nearest neighbors:

$x_{i}=\sum_{j=1}^{k}W\left(i,j\right)x_{j}$. The weights *W *∈ ℝ*^N×N ^*are estimated by minimizing the reconstruction error

$${\sum_{i=1}^{N}\left\|x_{i}-\sum_{j=1}^{k}W\left(i,j\right)x_{j}\right\|}^{2}$$

subject to *W *(*i, j*) = 0, if *x_i _*is not a neighbor of *x_j_*, and $\sum_{j=1}^{N}W\left(i,j\right)=1$. The last constraint ensures an invariance to translation next to rotation and rescaling. By minimizing

$$\epsilon \left(Y\right)={\sum_{i=1}^{N}\left\|y_{i}-\sum_{j=1}^{k}W\left(i,j\right)y_{j}\right\|}^{2}$$

,

the low-dimensional representation that best preserves the weights in the target space is chosen.

### Laplacian Eigenmaps

As well as LLE, Laplacian Eigenmaps (LEM) [38,39] are a local technique. Similar to Diffusion Maps, this method first constructs a neighborhood graph, weighted with values *W *(*i, j*) from the Gaussian kernel function. By minimizing a cost function

$$\epsilon \left(Y\right)=\sum_{ij}{\left\|y_{i}-y_{j}\right\|}^{2}W\left(i,j\right)$$

for neighbored *y_i_, y_j _*(*W *(*i, j*) = 0 otherwise), the distances between the low-dimensional representations are minimized and nearby samples *x_i_, x_j _*are highly weighted, and thus brought closer together. This way, Laplacian Eigenmaps implicitly enforces natural clusters in the data.

## Methods of Assessment

### Benchmark

**Figure 1:**
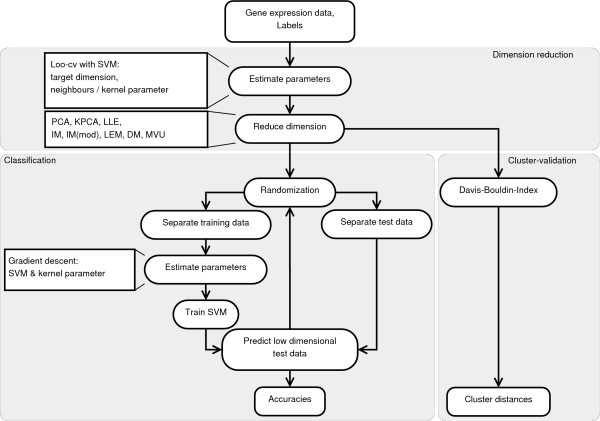
**Benchmark**. Our benchmark, consisting of three independent procedures. (1) Dimension reduction: Every dataset is mapped into a low-dimensional target space. All necessary parameters are determined by a loo-cv with a SVM. (2) Classification: Every dataset is classified by a SVM with Gaussian kernel during 100 randomization steps. A gradient descend procedure estimates all SVM parameters. (3) Cluster validation: The Davis-Bouldin-Index measures the distance between labeled clusters within the low-dimensional data.

Our benchmark (Figure 1) is divided into three parts. First, the studied dimension reduction methods were applied to the complete dataset. The low-dimensional datasets were then assessed by two different approaches, namely classification and cluster validation. To evaluate and compare the performance of each method, the classification accuracies of Support Vector Machines [40] (with Gaussian kernel) and the compactness and distance of clusters within the low-dimensional representations were used. In the following, each step of our benchmark is described in detail.

#### Datasets

The methods were tested on ten published microarray datasets as well as on simulated data. Each published dataset was divided into two classes according to a binary variable corresponding to the samples' disease status, the presence of certain molecular mutations or other sample characteristics as shown in Table 1. Since microarray data is technically provided with a more or less high level of noise, we reran the benchmark on the microarray datasets combined with normally distributed noise with zero mean and an increasing variance between 0 and 0.1. Before adding noise, all data was scaled to values between 0 and 1 to overcome the varying means and standard deviations of the datasets.

**Table 1:** Microarray datasets

Dataset	samples	features	class 1(#samples)	class 2(#samples)
1 Wang et al. - Breast cancer [50]	286	22.283	ER+(209)	ER-(77)

2 Verhaak et al. - Leukemia [51]	461	54.675	NPM1 pos.(140)	NPM1 neg.(321)

3 Haferlach et al. - Leukemia [52]	251	54.675	NPM1 pos.(138)	NPM1 neg.(113)

4 Haferlach et al. - Leukemia [52]	77	54.675	AML with t(8;21)(40)	AML with t(15;17)(37)

5 Golub et al. - Leukemia [53]	72	7.129	ALL(47)	AML(25)

6 Chiaretti et al. - Leukemia [54]	22	12.625	CLL stable(8)	CLL progressive(14)

7 Alizadeh et al. - Lymphoma [55]	38	18.432	Activated B-like DLBCL(17)	GC B-like DLBCL(21)

8 Nutt et al. - High-grade glioma [56]	50	12.625	Glioblastoma(28)	Anaplastic oligodendroglioma(22)

9 Alon et al. - Colon cancer [57]	62	2.000	Tumor(42)	Normal(20)

10 Singh et al. - Prostate cancer [58]	102	12.600	Tumor(52)	Normal(50)

Summary of all ten microarray gene expression datasets we used for testing the dimension reduction techniques. Here, we focus on the data by Wang et al., which represents best the results of the whole benchmark. Datasets 2-10 are shortly discussed in the supplement to this work. All datasets were separated into two classes according to two characteristics or the diagnosis of a disease.

The simulated data is based on a 50 sample dataset whose 10.000 gene expression values are normally distributed with zero mean and standard deviation one. The covariances of all genes are given by a block diagonal matrix with coefficients *ρ *= 0.2 within and *ρ *= 0 outside the blocks of size 50 × 50. To separate the data into two classes, between 10 and 500 genes were randomly chosen to be differentially expressed by adding a constant of 0.6 to the expression values of the first 25 samples. We generated 100 datasets for testing.

In the same manner as for the ten microarray datasets before, normally distributed noise with zero mean and an increasing variance between 0 and 0.2 was added to the simulated data. We repeated the benchmark on 50 of these noisy artificial datasets. The number of differential features was fixed to 300.

#### Dimension reduction

All dimension reduction techniques discussed here have one or two free parameters, that influence the embedding and the target dimension. Their determination was done by minimizing the error rate of a Support Vector Machine (SVM) within a leave-one-out cross-validation (loo-cv) schema: For *N *samples, the dataset was divided *N *times into a training and a test set. One sample was excluded for testing while the rest was taken for training. The average over all prediction accuracies gives an estimate of the SVMs' generalization error.

This procedure was repeated for every set of parameters within the following ranges:

Target dimensionality: 2 ≤ *d *≤ 15

Neighbors: 4 ≤ *k *≤ 16

Gaussian kernel: 1*e − *1 ≤ *σ *≤ 5*e*5

If the same loo-cv accuracies were achieved by using different parameter values for the target dimension, the lowest value was taken for reasons of a most simple representation. The same applies to the neighbor/kernel parameters.

After the loo-cv, the whole dataset was reduced in its dimension in an unsupervised manner, i.e. without consideration of class labels.

#### Classification

The first evidence for the quality of the different dimension reduction methods are the accuracies of a Support Vector Machine with Gaussian kernel.

The data was classified repeatedly during several randomization steps:

We randomly split the dataset a hundred times into a set to train the SVM and a test set for classification, and selected the median accuracy of all runs. Within the training set, a loo-cv was performed to determine the SVM parameters. For reasons of performance, a gradient descent procedure as proposed in [41] was used to minimize the loo-cv error. Every time during randomization, the training set consisted of two thirds of the original data and the test set of the remaining samples. The only constraint was to keep the balance between the number of samples in each class. Since SVMs do not restrict the dimension of the input data, the randomization results of the low-dimensional data can be compared to the high-dimensional original data, to see if more or less significant features got lost after the embedding.

#### Cluster validation

To measure the distances between the labeled clusters, we used the Davis-Bouldin-Index (DB-Index) [42]: Given *M *clusters *C_i _*(*i *= 1*, . . . , M *) and their centers $\mu_{i}=\frac{1}{\left|C_{i}\right|}\sum_{x\in C_{i}}x$,

$$d_{i}=\frac{1}{\left|C_{i}\right|}\sum_{x\in C_{i}}\left\|x-\mu_{i}\right\|$$

is the average distance of the samples in cluster *C_i _*to its center. While $R_{ij}=\frac{d_{i}+d_{j}}{\left\|\mu_{i}-\mu_{j}\right\|}$ reports the compactness of clusters *C_i _, C_j _*related to their distance, the DB-Index

$$DB=\frac{1}{M}\sum_{i=1}^{M}\max\left\{R_{ij}|1\leq j\leq M,i\neq j\right\}$$

averages the worst cases of the clusters' separations. One might expect well separated clusters to have smaller values close to one. In our case, the DB-Index was computed for fixed target space dimensions 2,3,5, and 10.

### Implementation details

The presented benchmark was implemented in Matlab 7.8.0 (R2009a). Furthermore, libsvm (version 2.89) [43] served as Support Vector Machine implementation, in conjunction with Automatic Model Selection for Kernel Methods (Apr 2005) [44]. The Dimensionality Reduction Toolbox (version 0.7 - Nov 2008) [45], Isomap package (Release 1 - Dec 2000) [46], LLE routine [45] and MVU implementation (version 1.3) [47] were used for dimension reduction. Because the Isomap and LLE routines performed best in our benchmark, we converted their Matlab implementations for the statistical programming language R [48]. The R-package 'RDRToolbox', also including a routine to compute the Davis-Bouldin-Index and our microarray gene expression data simulator, can be downloaded from [49] (see also Additional file 1).

## Results and Discussion

**Figure 2:**
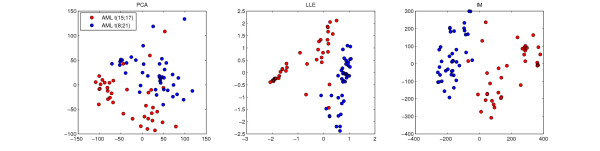
**Visualization**. Two dimensional visualization example of the Haferlach et al. Leukemia dataset. The LLE and Isomap embedding show more distinct clusters than the first two principal components of a PCA.

The following sections present the results for the Wang et al. Breast Cancer dataset, which represents best the results of the whole procedure. For the sake of simplicity, the visualization example in Figure 2 refers to the Haferlach et al. Leukemia dataset, which consists of fewer samples. Further detailed analysis of all other datasets is available in the supplement (see Additional file 2).

A linear approach like PCA is known to recover the true structure of data lying on or near a linear subspace of the high-dimensional input space. The following results show that the structure of microarray data is often too complex to be captured well in very low dimensional target spaces in a linear manner. Nonlinear methods, in particular LLE and Isomap, preserve more information in the data than the first few principle components of a PCA are able to cover.

### Classification

**Figure 3:**
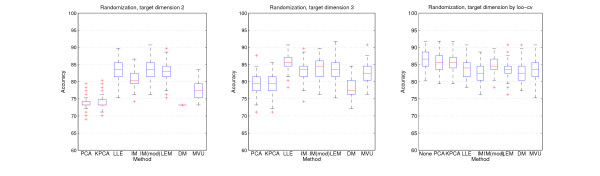
**Randomization accuracies**. Support Vector Machine classification accuracies of the Wang et al. breast cancer dataset. The data was randomized a hundred times for fixed target space dimensions two (left) and three (center) and for dimensions estimated by loo-cv (right). In the last case, the plot also shows the results for the original high-dimensional data without reduction. Especially in two and three dimensions, all nonlinear methods are superior to PCA.

The results of the randomization procedure are shown in Figure 3. In case of two and three dimensions, PCA performs worst, while all nonlinear methods, except Diffusion Maps, tend to retain the underlying structure of the data better in such low-dimensional target spaces.

Table 2 shows the parameters having the best loo-cv accuracies. The estimated target dimension was higher than ten in most cases. PCA and Kernel PCA result in the highest dimensions (14 and 15), while other methods like Laplacian Eigenmaps, MVU and Isomap worked best with less than ten dimensions. But classifications in two or three target dimensions often yield only slightly different accuracies. The classification accuracies on data with and without dimension reduction were often similar, even in two and three target dimensions.

**Table 2:** Parameter estimation

method	dim	neighbors/*σ*	loo-cv accuracy
PCA	14	-	87.4

KPCA	15	5e5	87.1

LLE	12	14	88.5

IM	8	10	85

IM(mod)	15	4	87.4

LEM	5	4	85.3

DM	13	5e5	84.3

MVU	5	14	85

All parameters of the dimension reduction techniques for the Wang et al. breast cancer dataset, estimated by leave-one-out cross-validation. PCA has no additional parameter, while KPCA and DM have a kernel parameter *σ *and IM, LEM and MVU take the number of neighbors as argument.

While all methods perform nearly even in higher dimensions, Isomap, LLE and Laplacian Eigenmaps performed best in two and three dimensions. Only on two of ten datasets (Alizadeh et. al and Singh et. al), PCA performed as well as other nonlinear methods like Isomap in two or three dimensional target spaces (see Supplemental Figures S18/S19, S27/S28). On all ten datasets considered together (see supplement), Diffusion Maps and Laplacian Eigenmaps produce more varying results and especially Diffusion Maps are very sensitive to the choice of the kernel parameter (see for example Figure 3, dimension two). But like Kernel PCA, they perform quite similar to PCA in most cases. MVU, which is based on Multidimensional Scaling like Isomap, is comparable to Isomap's good accuracies.

The initial publications on Isomap and MVU [25,27], covering text classification and face recognition, pointed out, that PCA might need higher dimensional target spaces than its nonlinear counterparts to lead to similar results. Since PCA only considers the variance in the data, it works best if those features, which are relevant for the class labeling, account most for the variance. Considering complex microarray data, the first two or three principal components were often not enough to cover the information necessary to sufficiently distinguish different classes within the data. This might prevent a well suited visualization, which is true to the original. LLE, Isomap and MVU, which classified best most of datasets, take advantage of overlapping local neighborhoods to create an image of the global geometry of the data. Although this approach may suffer from "holes" within the data (manifold), it proved more useful for accurate low-dimensional representations.

Well sampled datasets may overcome this issue of sparse data. But the Chiaretti et al. leukemia (22 samples), Alizadeh et al. lymphoma (38 samples) and Nutt et al. high-grade glioma dataset (50 samples) show that even with relatively few samples, a true to the original embedding is possible. The classification accuracies of most of the dimension reduction methods on these datasets (in ≥ 2 target dimensions) are comparable and sometimes even better than the accuracies on the high-dimensional data (see Supplemental Figures S15, S18, S21).

### Cluster validation

**Figure 4:**
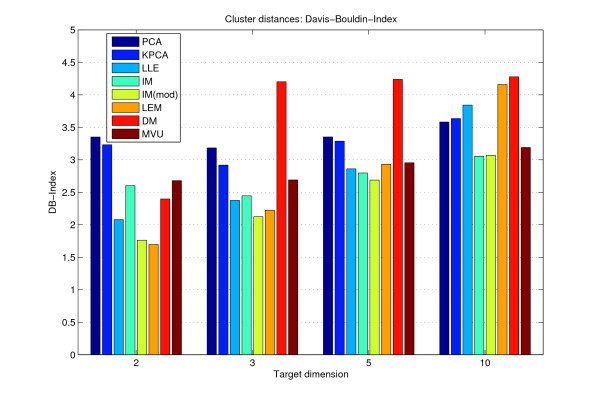
**Cluster validation**. Davis-Bouldin-Indices of the reduced Wang et al. breast cancer dataset for fixed target space dimensions 2,3,5 and 10. In most cases, the nonlinear methods produce more distinct clusters than PCA.

The cluster distances, presented in Figure 4, confirm the above conclusions. In two and three target dimensions, PCA results in worse scores than most nonlinear methods. DM performs the worst for more than two dimensions. With increasing target space dimension all methods converge, while the DB-Index itself increases as well. Although Laplacian Eigenmaps implicitly enforce natural clusters in the data, they show only slight different scores than e.g. LLE, which clusters best on most of the datasets. Just in case of ten target dimensions, LLE's and Laplacian Eigenmaps score remarkably worser on four of our ten datasets, while the other methods, including PCA, hold steady (see Supplemental Figures S9, S12, S21, S24). While Isomap might map well separated clusters to very close points, the slight modification of regarding nearest and farthest neighbors seems to correct this behavior on three datasets (Supplemental Figures S3, S6, S24), but performs similar or (much) worse otherwise (see for example Supplemental Figures S15, S18, S27). MVU scores similar to Isomap, but fails on three other datasets in two dimensional target spaces (Supplemental Figures S6, S15, S18).

Because LLE and Isomap performed best on most of the datasets during classification and cluster validation, Figure 2 compares their two dimensional embedding of the Haferlach et al. Leukemia dataset to the first two principal components of a PCA. All three visualizations clearly show two clusters of AML patients with t(15;17) and t(8;21) respectively. But LLE and Isomap distinguish both classes best, while in the PCA embedding three more t(15;17) samples lie between samples of the other class. Since LLE and Isomap both map more samples correctly, there seems to be more information within the data, that the first two PCA components fail to preserve. On closer inspection, the common three t(15;17) outliers, that are in between or closest to t(8;21) samples in all three visualizations, are always the same samples #44 and #46 #57. Another visualization example of the Alon et al. Colon Cancer dataset with all eight dimension reduction techniques can be seen in Supplemental Figures S1 and S2.

### Noise evaluation

**Figure 5:**
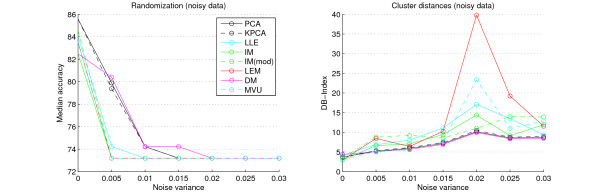
**Noise evaluation**. Randomization accuracies (left) and cluster-indices (right) for the Wang et al. breast cancer dataset combined with normally distributed noise with zero mean and different variances. PCA and DM react most stable on noise. All other methods lead to varying accuracies and cluster scores.

The tests on artificially noised microarray datasets reveal, that PCA, Kernel PCA and Diffusion Maps are most robust on noisy data (Figure 5). But the differences are less strong and the results more variable than for the classification and cluster validation without adding noise. The sensitivity to noise of all methods strongly depends on the given class labels and associated features, and thus leads to varying results between all ten datasets (see supplement). While Diffusion Maps are known to be robust to noise [14], all other nonlinear methods, especially Isomap and its modification, suffer most from unstructured data and lead to strongly varying cluster scores.

### Simulated data

**Figure 6:**
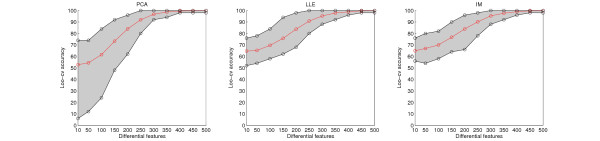
**Simulated data**. Leave-one-out cross-validation accuracies of the simulated datasets with increasing differential features. The red line marks the average accuracy of all 100 generated datasets within the 95% quantile. In two dimensional target spaces, LLE an IM capture more of the underlying structure of the data with much fewer significant features than PCA.

Since LLE and Isomap performed best in the first two tests, the classifications on simulated data refer only to these methods. In all three cases, we fixed a two dimensional target space. Figure 6 shows that the results of the loo-cv on real microarray datasets can be reproduced on simulated data. With only few differential features, LLE and Isomap already capture more of the structure of the data than PCA. It takes more than 150 (of overall 10.000) differential features for PCA to perform nearly even. Furthermore, for less than 200 differential features, the accuracies of PCA are spreading much stronger, while LLE and Isomap give more stable results. The findings for three target dimensions are similar to the two dimensional case and can be seen in the supplement (Supplemental Figure S30).

The benchmark with noisy simulated data, however, confirms the results of the noise evaluation for the ten microarray datasets. Supplemental Figures S31 and S32 show for two and three target dimensions, that PCA performs more robust than LLE and Isomap for both, classification and cluster validation, when noise within the data increases. These conclusions hold true for noisy data with a larger variance, since PCA, LLE and Isomap are invariant to multiplication of the data with a scalar.

### Statistical hypothesis test

In Table 3, we compare all results by applying the Wilcoxon signed-rank test on the accuracies and cluster scores for two dimensional data representations. We tested the null hypothesis, that the median of the differences between PCA and each of the nonlinear methods is equal to zero. This way, we computed the p-values of 14 paired samples. The p-values were not adjusted for multiple testing. Isomap and LLE show the most significant results in accuracy and clustering with p-values 0.0078 and 0.0273 respectively. Diffusion Maps led to results most similar to PCA.

**Table 3:** Wilcoxon signed-rank test (p-values)

**PCA compared to ..**.	Accuracies(dim 2)	DB-Index(dim 2)
KPCA	0.1562	0.3223

LLE	0.0195	**0.0273**

IM	**0.0078**	0.1055

IM(mod)	0.0547	0.2324

LEM	0.1953	0.3750

DM	0.5000	0.8457

MVU	0.1953	0.7695

The p-values of the Wilcoxon signed-rank test based on the null hypothesis, that PCA yields results similar to each other nonlinear method. The median randomization accuracies and cluster scores (both for two target space dimensions) of all ten datasets served as input to the tests.

### Runtime

The computational complexity and memory requirements for all dimension reduction methods except MVU are equal, as shown in Supplemental Table S3. However, we observed differences in runtime between the methods due to different constant factors. Table 4 lists the runtime of all seven methods in seconds for the smallest dataset (Chiaretti et al. leukemia dataset, 22 samples) and the largest dataset (Verhaak et al. leukemia dataset, 461 samples). The target dimensionality was set to two. The embeddings were computed on an AMD Opteron processor with 2 GHz.

**Table 4:** Runtime

	PCA	KPCA	LLE	IM	LEM	DM	MVU
Chiaretti et al.	0.09 s	0.03 s	0.14 s	0.04 s	0.04 s	0.16 s	0.25 s

Verhaak et al.	9.4 s	12.7 s	21.9 s	14 s	15.2 s	13.2 s	2 hrs

Runtime in seconds of all seven dimension reduction techniques on the Chiaretti et al. leukemia dataset (*N *= 22, *D *= 12.625, *d *= 2, *k *= 10, *σ *= 10) and the Verhaak et al. leukemia dataset (*N *= 461, *D *= 54.675, *d *= 2, *k *= 10, *σ *= 100). In the latter case, the runtime of MVU is given in hours.

The runtime of all nonlinear methods (Kernel PCA, Isomap, LLE, LEM, DM, MVU) depends on the number of samples. Even for relatively large microarray datasets (461 samples in this case), runtimes between 9.4 and 21.9 seconds are acceptable for visualization purposes. Only the solution of a semidefinite program in the MVU algorithm takes two hours. For all methods, the computing time for datasets with more common sample sizes (≤ 50) is less than a second.

## Conclusions

Classifications on high and low-dimensional data showed, that the most significant information within microarray data can be captured quite well in very few dimensions compared to the thousands of features of the original data.

Our benchmark further revealed significant shortcomings of PCA in two and three dimensional target spaces and brought out two nonlinear methods, that distinguished most from PCA. Especially the performances of Locally Linear Embedding and Isomap in classification and cluster validation make them well suited alternatives to the classic, linear approach of PCA.

## Authors' contributions

CB designed and implemented the benchmark and wrote the paper. HK and CR analyzed results and helped writing the manuscript. MD and XJ contributed to the benchmark design. All authors read and approved the final manuscript.

## Supplementary Material

Additional file 1 **R-package**. RDRToolbox_1.0.0.tar.gz: A package for nonlinear dimension reduction using the Isomap and LLE algorithm. It also includes a routine for computing the Davis-Bouldin-Index for cluster validation, a plotting tool and a data generator for microarray gene expression data and for the Swiss Roll dataset. Click here for file

Additional file 2 **Supplement**. Supplement.pdf: Contains information about preprocessing of the data, a discussion of the computational complexity of each dimension reduction method, further classification, cluster validation and noise evaluation results of nine other microarray datasets and further classification and randomization results for simulated datasets. Click here for file
