# Supplementary material for: Comparative study of unsupervised dimension reduction techniques for the visualization of microarray gene expression data
Source: BMC Bioinformatics. 2010 Nov 18;11:567. doi: 10.1186/1471-2105-11-567 (PMC2998530; doi:10.1186/1471-2105-11-567)
Supplement: Additional file 1 — R-package. RDRToolbox_1.0.0.tar.gz: A package for nonlinear dimension reduction using the Isomap and LLE algorithm. It also includes a routine for computing the Davis-Bouldin-Index for cluster validation, a plotting tool and a data generator for microarray gene expression data and for the Swiss Roll dataset. [file 1471-2105-11-567-S1.GZ › RDRToolbox/inst/doc/vignette.pdf]

RDRToolbox
A package for nonlinear dimension reduction with Isomap and LLE.

Christoph Bartenhagen

October 18, 2010

Contents

1 Introduction **1**
1.1 Loading the package 2

2 Datasets **2**
2.1 Swiss Roll 2
2.2 Simulating microarray gene expression data 3

3 Dimension Reduction **3**
3.1 Locally Linear Embedding 3
3.2 Isomap 4

4 Plotting **5**

5 Cluster distances **7**

6 Example **8**

1 Introduction

High-dimensional data, like for example microarray gene expression data, can often be reduced to only a few significant features without losing important information. Dimension reduction methods perform a mapping between an original high-dimensional input space and a target space of lower dimensionality. A 'good' dimension reduction technique should preserve most of the significant information and generate data with similar characteristics like the high-dimensional original. For example, clusters should also be found within the reduced data, preferably more distinct.
This package provides the Isomap and Locally Linear Embedding algorithm for nonlinear dimension reduction. Nonlinear means in this case, that the methods were designed with respect to data lying on or near a nonlinear submanifold in the higher dimensional input space and perform a nonlinear mapping.
Further, both algorithms belong to the so called feature extraction methods, which in contrast to feature selection methods combine the information from all features. These approaches are often most suited for low-dimensional representations of the whole data.
For cluster validation purposes, the package also includes a routine for computing the Davis-Bouldin-Index.

Further, a plotting tool visualizes two and three dimensional data and, where appropriate, its clusters. For testing, the well known Swiss Roll dataset can be computed and a data generator simulates microarray gene expression data of a given (high) dimensionality.

1.1 Loading the package

The package can be loaded into R by typing

```
> library(RDRToolbox)
```

into the R console. To create three dimensional plots, the toolbox requires the package *rgl*. Otherwise, only two dimensional plots will be available. Furthermore, the package *MASS* has to be installed for gene expression data simulation (see section 2.2).

2 Datasets

In general, the dimension reduction, cluster validation and plot functions in this toolbox expect the data as $N \times D$ matrix, where N is the number of samples and D the dimension of the input data (number of features). After processing, the data is returned as $N \times d$ matrix, for a given target dimensionality d ($d < D$).
But before describing the dimension reduction methods, this section shortly covers two ways of generating a dataset.

2.1 Swiss Roll

The function `SwissRoll` computes and plots the three dimensional Swiss Roll dataset of a given size `N` and `Height`. If desired, it uses the package *rgl* to visualize the Swiss Roll as a rotatable 3D scatterplot. The following example computes and plots a Swiss Roll dataset containing 1.000 samples (argument `Height` set to 30 by default):

```
> swissData = SwissRoll(N = 1000, Plot = TRUE)
```

See figure 1 below for the plot.

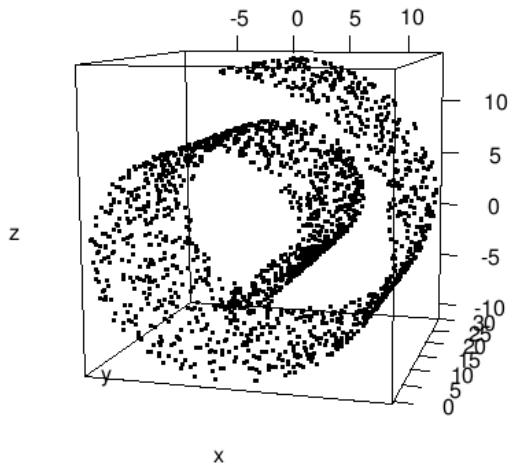

Figure 1: The three dimensional SwissRoll dataset.

2.2 Simulating microarray gene expression data

The function `generateData` is a simulator for gene expression data, whose values are normally distributed values with zero mean. The covariance structure is given by a configurable block-diagonal matrix. To simulate differential gene expression between the samples, the expression of a given number of features (parameter `diffgenes`) of some of the samples will be increased by adding a given factor `diff` (0.6 by default). The parameter `diffsamples` controls how many samples have higher expression values compared to the rest (by default, the values of half of the total number of samples will be increased).
The simulator generates two labelled classes:

label 1: samples with differentially expressed genes.

label -1: samples without differentially expressed genes.

Finally, `generateData` returns a list containing the data and its class labels.

The following example computes a `dim`=1.000 dimensional dataset where 10 of 20 samples contain `diffgenes`=100 differential features:

```
> sim = generateData(samples = 20, genes = 1000,
     diffgenes = 100, diffsamples = 10)
> simData = sim[[1]]
> simLabels = sim[[2]]
```

The covariance can be modified using the arguments `cov1`, `cov2`, to set the covariance within and between the blocks of size `blocksize` × `blocksize` of the block-diagonal matrix:

```
> sim = generateData(samples = 20, genes = 1000,
     diffgenes = 100, cov1 = 0.2, cov2 = 0, blocksize = 10)
> simData = sim[[1]]
> simLabels = sim[[2]]
```

3 Dimension Reduction

3.1 Locally Linear Embedding

Locally Linear Embedding (LLE) was introduced in 2000 by Roweis, Saul and Lawrence [1, 2]. It preserves local properties of the data by representing each sample in the data by a linear combination of its k nearest neighbours with each neighbour weighted independently. LLE finally chooses the low-dimensional representation that best preserves the weights in the target space.

The function `LLE` performs this dimension reduction for a given dimension `dim` and neighbours `k`. The following examples compute a two and a three dimensional LLE embedding of the simulated 1.000 dimensional dataset seen in the example in section 2.2 using `k`=10 and 5 neighbours:

```
> simData_dim3_lle = LLE(data = simData, dim = 3,
     k = 10)
> head(simData_dim3_lle)
```

```
           [,1]       [,2]       [,3]
[1,] 1.63405485 -1.2035617 -0.3427752
[2,] 0.59568282 -0.9892234  0.2269532
[3,] 0.51071424  1.3924488 -0.6739528
```

```
[4,] 0.12068684  0.6953508  0.5026695
[5,] 0.07603239  0.9261577 -0.4413328
[6,] 1.22035061  0.0906943  1.8496204
```

```
> simData_dim2_lle = LLE(data = simData, dim = 2,
     k = 5)
> head(simData_dim2_lle)
```

```
           [,1]        [,2]
[1,] -0.2148390 -0.68591623
[2,] -1.1682295 -1.56907087
[3,] -0.7004933  0.83351524
[4,] -1.2987830 -0.05152696
[5,] -1.3254800  0.19330016
[6,] -1.0511688 -0.91381928
```

3.2 Isomap

Isomap (IM) is a nonlinear dimension reduction technique presented by Tenenbaum, Silva and Langford in 2000 [3, 4]. In contrast to LLE, it preserves global properties of the data. That means, that geodesic distances between all samples are captured best in the low dimensional embedding. This implementation uses Floyd's Algorithm to compute the neighbourhood graph of shortest distances, when calculating the geodesic distances.

The function `Isomap` performs this dimension reduction for a given vector of dimensions `dims` and neighbours `k`. It returns a list of low-dimensional datasets according to the given dimensions.

The following example computes a two dimensional Isomap embedding of the simulated 1.000 dimensional dataset seen in the example in section 2.2 using `k`=10 neighbours:

```
> simData_dim2_IM = Isomap(data = simData, dims = 2,
     k = 10)
> head(simData_dim2_IM$dim2)
```

```
           [,1]       [,2]
[1,]  -9.045206  42.188393
[2,]   7.257905  13.695555
[3,] -47.725052 -16.240927
[4,] -32.975175 -16.609374
[5,]  -9.577050  -3.901239
[6,] -14.962417  21.719676
```

Setting the argument `plotResiduals` to `TRUE`, `Isomap` shows a plot with the residuals between the high- and the low-dimensional data (here, for target dimensions 1-10). It can help estimating the intrinsic dimension of the data:

```
> simData_dim1to10_IM = Isomap(data = simData, dims = 1:10,
     k = 10, plotResiduals = TRUE)
```

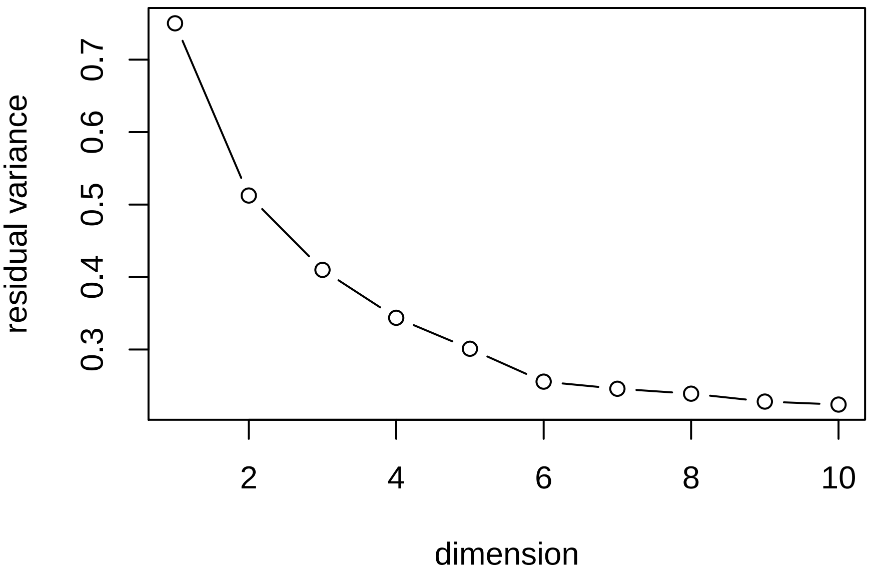

This implementation further includes a modified version of the original Isomap algorithm, which respects nearest and farthest neighbours. The next call of `Isomap` varies the upper example by setting the argument `mod`:

```
> Isomap(data = simData, dims = 2, mod = TRUE, k = 10)
```

4 Plotting

The function `plotDR` creates two and three dimensional plots of (labelled) data. It uses the library *rgl* for rotatable 3D scatterplots. The data points are coloured according to given class labels (max. six classes when using default colours). A legend will be printed in the R console by default. The parameter `legend` or the R command `legend` can be used to add a legend to a two dimensional plot (a legend for three dimensional plots is not supported).

The first example plots the two dimensional embedding of the artificial dataset from section 2.2:

```
> plotDR(data = simData_dim2_lle, labels = simLabels)

  class colour
1    -1  black
2     1    red
```

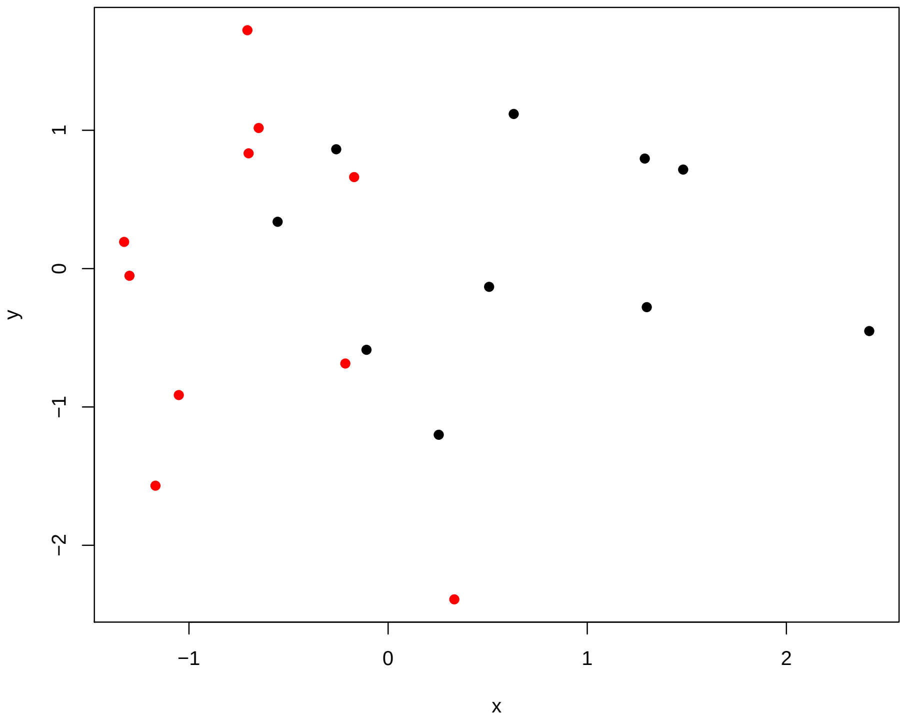

By specifying the arguments `axesLabels` and `text`, labels for the axes and for each data point (sample) respectively can be added to the plot:

```
> samples = c(rep("class 1", 10), rep("class 2",
     10))
> labels = c("first component", "second component")
> plotDR(data = simData_dim2_lle, labels = simLabels,
     axesLabels = labels, text = samples)

  class colour
1    -1  black
2     1    red
```

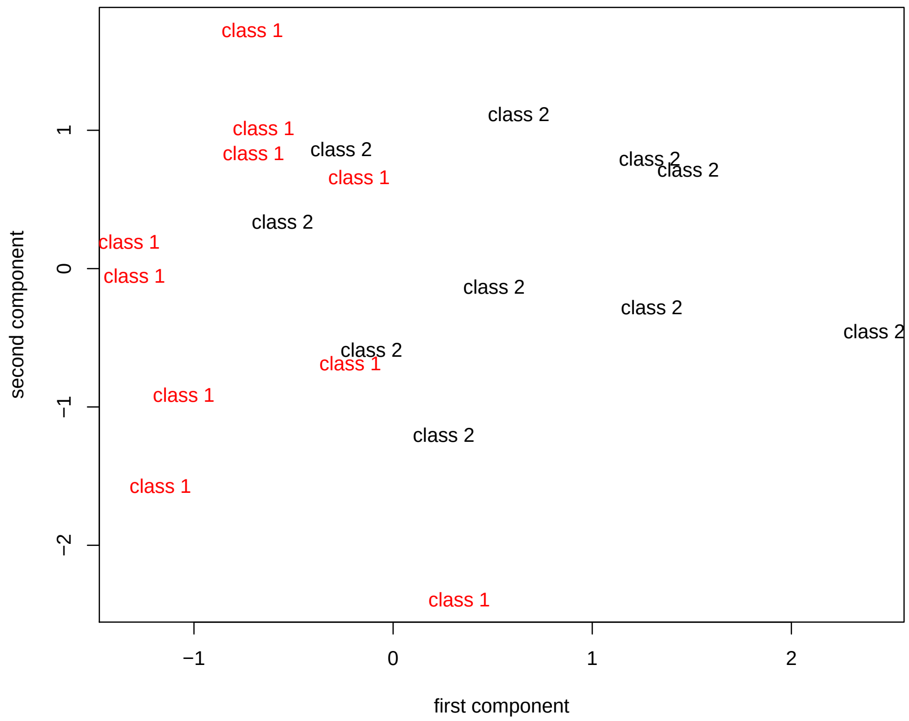

A plot of a three dimensional LLE embedding of a 1.000 dimensional dataset is given by

```
> plotDR(data = simData_dim3_lle, labels = simLabels)
```

5 Cluster distances

The function DBIndex computes the Davis-Bouldin-Index (DB-Index) for cluster validation purposes. The index relates the compactness of each cluster to their distance: To compute a clusters' compactness, this version uses the Euclidean distance to determine the mean distances between the samples and the cluster centres. The distance of two clusters is given by the distance of their centres. The smaller the index the better. Values close to 1 or smaller indicate well separated clusters.

The following example computes the DB-Index of a 50 dimensional dataset with 20 samples separated into two classes/clusters:

```
> d = generateData(samples = 20, genes = 50, diffgenes = 10,
     blocksize = 5)
> DBIndex(data = d[[1]], labels = d[[2]])
[1] 3.846423
```

As the two dimensional plots in section 4 anticipated, the low-dimensional LLE dataset has quite well separated clusters. Accordingly, the DB-Index is low:

```
> DBIndex(data = simData_dim2_lle, labels = simLabels)
[1] 1.594522
```

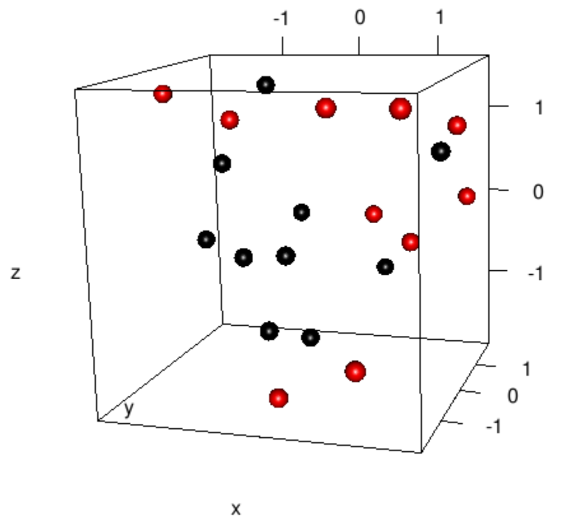

Figure 2: Three dimensional simulated microarray dataset (reduced from formerly 1.000 dimensions by LLE)

6 Example

This section demonstrates the dimension reduction workflow for the publicly available the Golub et al. leukemia dataset.
The data is available as R package and can be loaded via

```
> library(golubEsets)
> data(Golub_Merge)
```

The dataset consists of 72 samples, divided into 47 ALL and 25 AML patients, and 7129 expression values. In this example, we compute a two dimensional LLE and Isomap embedding and plot the results.

At first, we extract the features and class labels:

```
> golubExprs = t(exprs(Golub_Merge))
> labels = pData(Golub_Merge)$ALL.AML
> dim(golubExprs)

[1]   72 7129

> show(labels)

 [1] ALL ALL ALL ALL ALL ALL ALL ALL ALL ALL ALL ALL ALL ALL
[15] ALL ALL ALL ALL ALL ALL AML AML AML AML AML AML AML AML
[29] AML AML AML AML AML AML ALL ALL ALL ALL ALL ALL ALL ALL
[43] ALL ALL ALL ALL ALL ALL ALL ALL ALL ALL ALL ALL ALL ALL
[57] ALL ALL ALL ALL ALL AML AML AML AML AML AML AML AML AML
[71] AML AML
Levels: ALL AML
```

The residual variance of Isomap can be used to estimate the intrinsic dimension of the dataset:

```
> Isomap(data = golubExprs, dims = 1:10, plotResiduals = TRUE,
     k = 5)
```

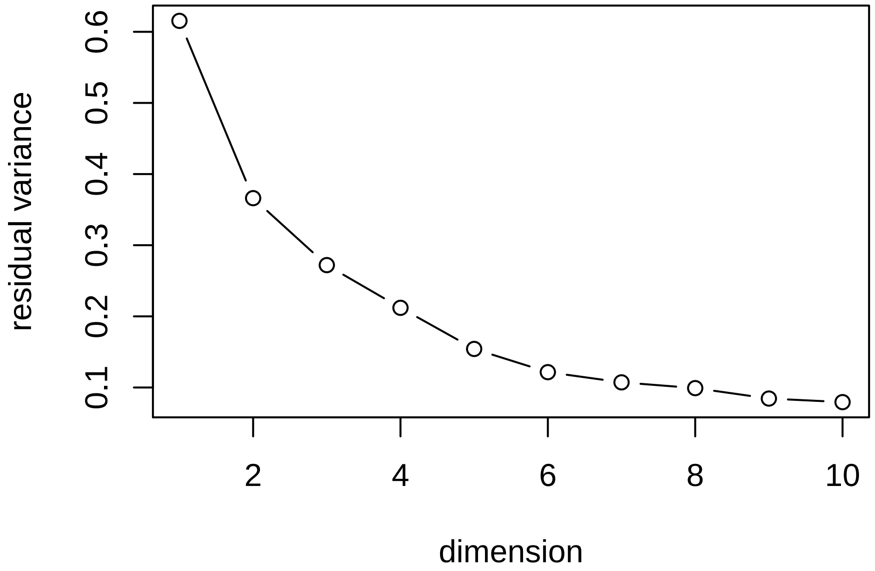

Regarding the dimensions for which the residual variances stop to decrease significantly, we can expect a low intrinsic dimension of two or three and therefore, a visualization true to the structure of the original data.

Next, we compute the LLE and Isomap embedding for two target dimensions:

```
> golubIsomap = Isomap(data = golubExprs, dims = 2,
     k = 5)
> golubLLE = LLE(data = golubExprs, dim = 2, k = 5)
```

The Davis-Bouldin-Index shows, that the ALL and AML patients are well separated into two clusters:

```
> DBIndex(data = golubIsomap$dim2, labels = labels)

[1] 1.131335

> DBIndex(data = golubLLE, labels = labels)

[1] 1.172901
```

Finally, we use `plotDR` to plot the two dimensional data:

```
> plotDR(data = golubIsomap$dim2, labels = labels,
     axesLabels = c("", ""), legend = TRUE)
> title(main = "Isomap")
```

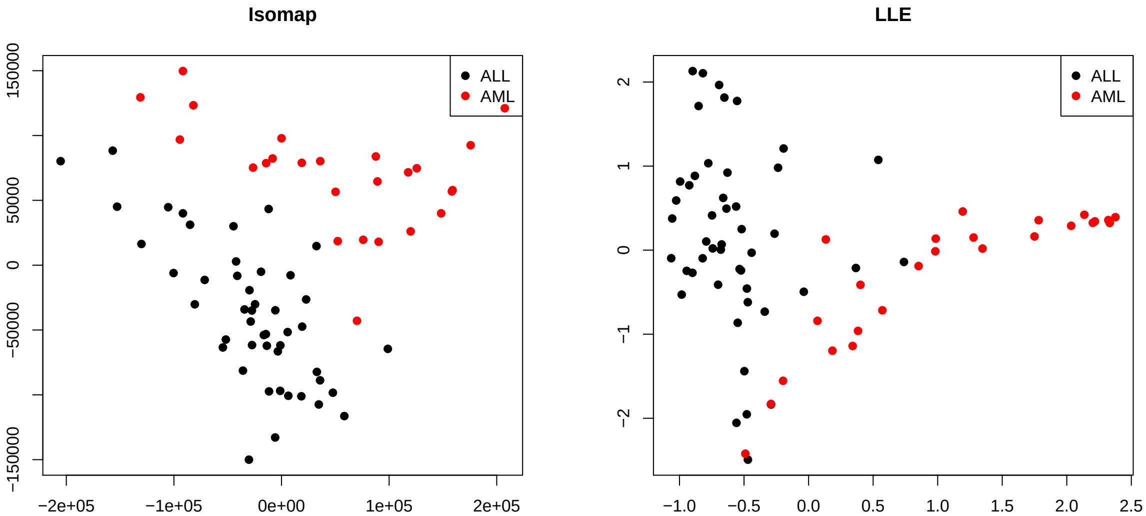

Figure 3: Two dimensional embedding of the Golub et al. leukemia dataset (Left: Isomap; Right: LLE).

```
> plotDR(data = golubLLE, labels = labels, axesLabels = c("",
      ""), legend = TRUE)
> title(main = "LLE")
```

Both visualizations, using either Isomap or LLE, show distinct clusters of ALL and AML patients, although the cluster overlap less in the Isomap embedding. This is consistent with the DB-Index, which is very low for both methods, but slightly higher for LLE.

A three dimensional visualization can be generated in the same manner and is best analyzed interactively within R.

References

[1] S. T. Roweis and L. K. Saul. Nonlinear dimensionality reduction by locally linear embedding. *Science*, 290(5500):2323–2326, 2000.

[2] L. K. Saul and S. T. Roweis. Think globally, fit locally: unsupervised learning of low dimensional manifolds. *Journal of Machine Learning Research*, 4:119–155, June 2003.

[3] V. D. Silva and J. B. Tenenbaum. Global versus local methods in nonlinear dimensionality reduction. In *Advances in Neural Information Processing Systems 15*, pages 705–712. MIT Press, 2003.

[4] J. B. Tenenbaum, V. de Silva, and J. C. Langford. A global geometric framework for nonlinear dimensionality reduction. *Science*, 290(5500):2319–2323, December 2000.
