# Supplementary figures and images for: Comparative study of unsupervised dimension reduction techniques for the visualization of microarray gene expression data
Source: BMC Bioinformatics. 2010 Nov 18;11:567. doi: 10.1186/1471-2105-11-567 (PMC2998530; doi:10.1186/1471-2105-11-567)

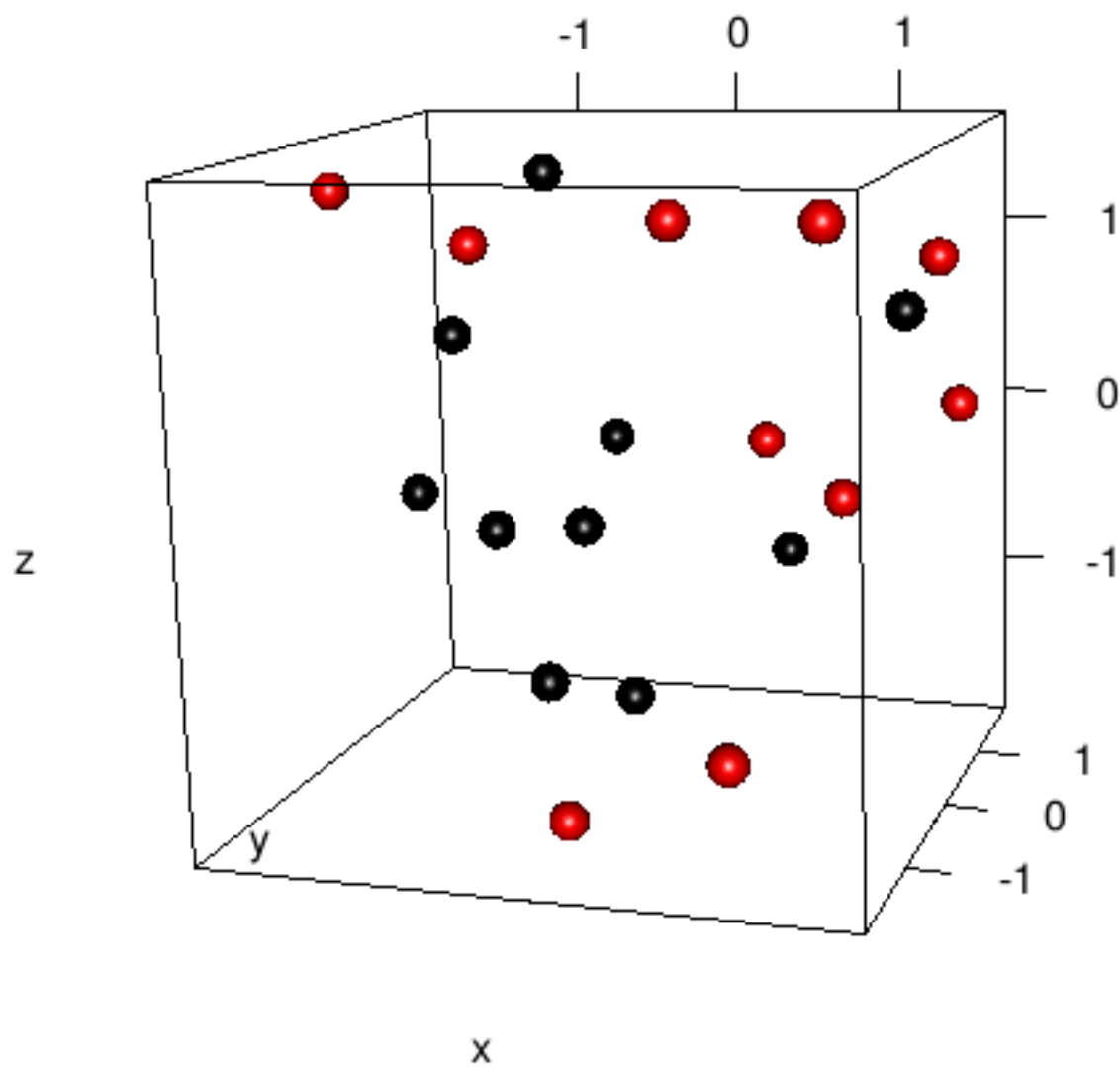

Supplement: Additional file 1 — R-package. RDRToolbox_1.0.0.tar.gz: A package for nonlinear dimension reduction using the Isomap and LLE algorithm. It also includes a routine for computing the Davis-Bouldin-Index for cluster validation, a plotting tool and a data generator for microarray gene expression data and for the Swiss Roll dataset. [file 1471-2105-11-567-S1.GZ › RDRToolbox/inst/doc/plot3D.pdf]

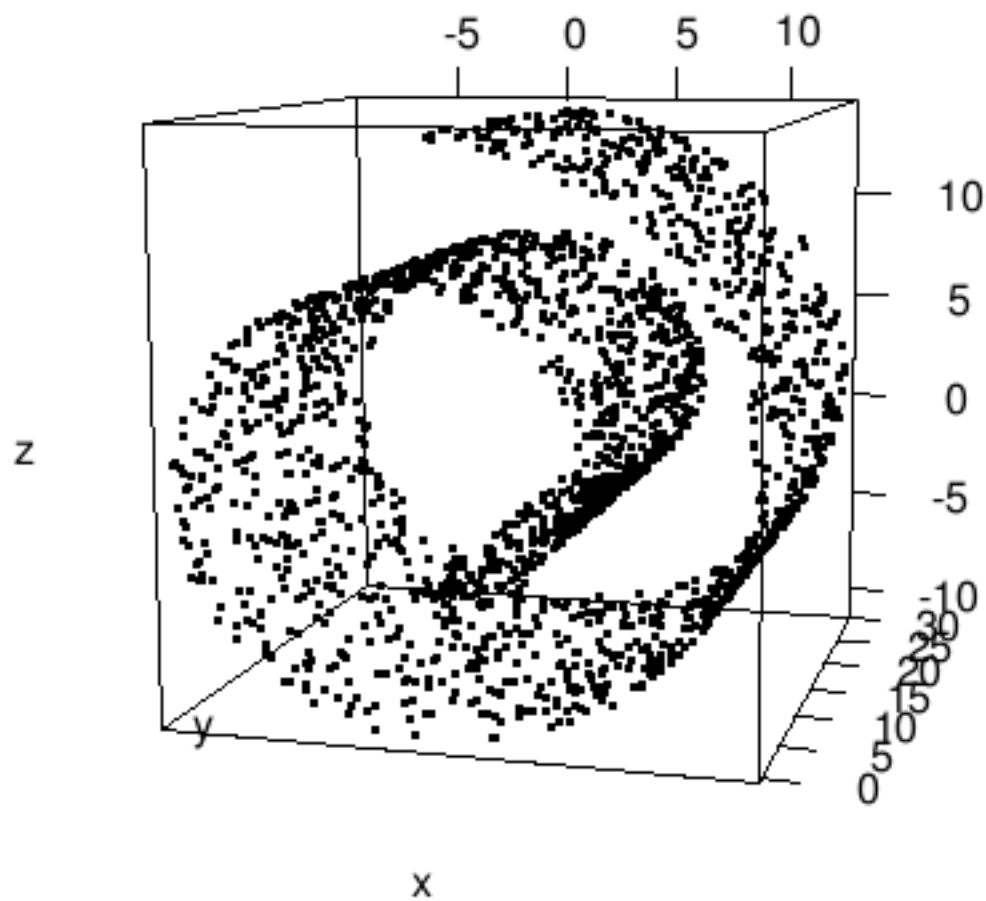

Supplement: Additional file 1 — R-package. RDRToolbox_1.0.0.tar.gz: A package for nonlinear dimension reduction using the Isomap and LLE algorithm. It also includes a routine for computing the Davis-Bouldin-Index for cluster validation, a plotting tool and a data generator for microarray gene expression data and for the Swiss Roll dataset. [file 1471-2105-11-567-S1.GZ › RDRToolbox/inst/doc/SwissRoll.pdf]
