# Supplementary material for: Comparative study of unsupervised dimension reduction techniques for the visualization of microarray gene expression data
Source: BMC Bioinformatics. 2010 Nov 18;11:567. doi: 10.1186/1471-2105-11-567 (PMC2998530; doi:10.1186/1471-2105-11-567)
Supplement: Additional file 2 — Supplement. Supplement.pdf: Contains information about preprocessing of the data, a discussion of the computational complexity of each dimension reduction method, further classification, cluster validation and noise evaluation results of nine other microarray datasets and further classification and randomization results for simulated datasets. [file 1471-2105-11-567-S2.PDF]

Comparative study of unsupervised dimension reduction techniques for the visualization of microarray gene expression data (Supplemental information)

Christoph Bartenhagen*[1], Hans-Ulrich Klein[1], Christian Ruckert[1], Xiaoyi Jiang[2] and Martin Dugas[1]

[1]Department of Medical Informatics and Biomathematics, University of Münster, Domagkstraße 9, 48149 Münster, Germany
[2]Department of Computer Science, University of Münster, Einsteinstrasse 62, 48149 Münster, Germany

Email: Christoph Bartenhagen*- christoph.bartenhagen@ukmuenster.de; Hans-Ulrich Klein - h.klein@uni-muenster.de; Christian Ruckert - cruckert@uni-muenster.de; Xiaoyi Jiang - xjiang@uni-muenster.de; Martin Dugas - dugas@uni-muenster.de;

*Corresponding author

Contents

1 Datasets and preprocessing 1

2 Computational complexity 2

3 Visualization 3

4 Verhaak et al. - Leukemia dataset 5

5 Haferlach et al. - Leukemia dataset (NPM1 labeling) 6

6 Haferlach et al. - Leukemia dataset (AML labeling) 7

7 Golub et al. - Leukemia dataset 8

8 Chiaretti et al. - Leukemia dataset 9

9 Alizadeh et al. - Lymphoma dataset 10

10 Nutt et al. - High-grade glioma dataset 11

11 Alon et al. - Colon cancer dataset 12

12 Singh et al. - Prostate cancer dataset 13

13 Simulated data - Randomization for three target dimensions 14

14 Simulated data - Noise evaluation 14

1 Datasets and preprocessing

In this supplement, we list the SVM classification results and Davis-Bouldin-Indices for cluster validation of nine of the following microarray datasets (2-10). The Wang et al. dataset has been discussed in the original article.

	samples	features	class 1(#samples)	class 2(#samples)
1 Wang et al. - Breast cancer [1]	286	22.283	ER+(209)	ER-(77)
2 Verhaak et al. - Leukemia [2]	461	54.675	NPM1 pos.(140)	NPM1 neg.(321)
3 Haferlach et al. - Leukemia [3]	251	54.675	NPM1 pos.(138)	NPM1 neg.(113)
4 Haferlach et al. - Leukemia [3]	77	54.675	AML with t(8;21)(40)	AML with t(15;17)(37)
5 Golub et al. - Leukemia [4]	72	7.129	ALL(47)	AML(25)
6 Chiaretti et al. - Leukemia [5]	22	12.625	CLL stable(8)	CLL progressive(14)
7 Alizadeh et al. - Lymphoma [6]	38	18.432	Activated B-like DLBCL(17)	GC B-like DLBCL(21)
8 Nutt et al. - High-grade glioma [7]	50	12.625	Glioblastoma(28)	Anaplastic oligodendroglioma(22)
9 Alon et al. - Colon cancer [8]	62	2.000	Tumor(42)	Normal(20)
10 Singh et al. - Prostate cancer [9]	102	12.600	Tumor(52)	Normal(50)

Table S1: Summary of all ten microarray gene expression datasets we used for testing the dimension reduction techniques. All datasets were separated into two classes according to two characteristics or the diagnosis of a disease.

The datasets were preprocessed as shown in table S2 below. Except for the Alizadeh et. al Lymphoma dataset, we used the normalized data as provided by the authors.

	preprocessing/normalization	Availability
1 Wang et al. - Breast cancer [1]	Image was globally scaled to a target intensity of 600	GEO: GSE2034
2 Verhaak et al. - Leukemia [2]	MAS5 with global scaling to target value 100, then values <= 30 were set to 30 followed by a log2 transformation	GEO: GSE6891
3 Haferlach et al. - Leukemia [3]	Robust Multichip Average algorithm (RMA, see R package affy version 1.20.2)	GEO: GSE15434
4 Haferlach et al. - Leukemia [3]	Robust Multichip Average algorithm (RMA, see R package affy version 1.20.2)	GEO: GSE15434
5 Golub et al. - Leukemia [4]	Intensity values were rescaled such that overall intensities for each sample are equivalent	R package golubEsets.
6 Chiaretti et al. - Leukemia [5]	Robust Multichip Average algortihm with probe sequence information (GCRMA, see R package gcrma)	R package CLL
7 Alizadeh et al. - Lymphoma [6]	Lowess normalization and background subtraction	available at [10]
8 Nutt et al. - High-grade glioma [7]	Linear scaling such that mean array intensity for "present" genes (according to Affymetrix) was identical for all scans	available at [11]
9 Alon et al. - Colon cancer [8]	2000 genes with highest minimal intensity were picked, data is otherwise unprocessed	available at [12]
10 Singh et al. - Prostate cancer [9]	Expression were files scaled to reference file (having median value of expression) by the mean average difference for all genes	available at [13]

Table S2: Preprocessing/normalization and availability of all datasets we used for testing.

2 Computational complexity

The computational and memory complexity of all seven dimension reduction methods, as shown in Table S3 below mainly depends on the number of samples N. The size of the neighbourhood k only matters for MVU.

method	computational costs	memory
PCA	$O(N^3)$ or $O(D^3)$	$O(N^2)$ or $O(D^2)$
KPCA	$O(N^3)$	$O(N^2)$
LLE	$O(N^3)$	$O(N^2)$
IM	$O(N^3)$	$O(N^2)$
LEM	$O(N^3)$	$O(N^2)$
DM	$O(N^3)$	$O(N^2)$
MVU	$O((kN)^3)$	$O((kN)^3)$

Table S3: Computational complexity of all seven dimension reduction methods techniques discussed in this thesis. Influencing parameters are the number of samples N, the number of features D and the number of neighbours k.

Except for MVU, the last step of computing the eigenvalues and eigenvectors of a $N \times N$ weight (IM, DM), cost (LLE), Laplacian (LEM), covariance (PCA) or kernel matrix (KPCA) results in an overall complexity of $O(N^3)$ (using e.g. the QR algorithm). In addition, Isomap makes use of a shortest path algorithm (Floyd's algorithm) on a graph with N vertices that also takes $O(N^3)$ computations. Storing the matrices yield memory complexities of $O(N^2)$. MVU needs to solve a semidefinite program with kN constraints, whose computational and memory complexity is $O((kN)^3)$.

PCA, usually computing the covariances between all D features, requires $O(D^3)$ computations. In case of microarray gene expression data, where $D \gg N$, the embedding was based on the squared Euclidean distances between the samples and results in complexities comparable to most of the other nonlinear methods.

Special methods for sparse eigenvalue problems can be applied to local methods like LLE and Laplacian Eigenmaps to yield nearly quadratic complexities.

3 Visualization

Figures S1 and S2 show eight different two dimensional embeddings of the Alon et al. colon cancer dataset (62 samples). The classification and cluster validation results for this dataset can be seen below on page 12.

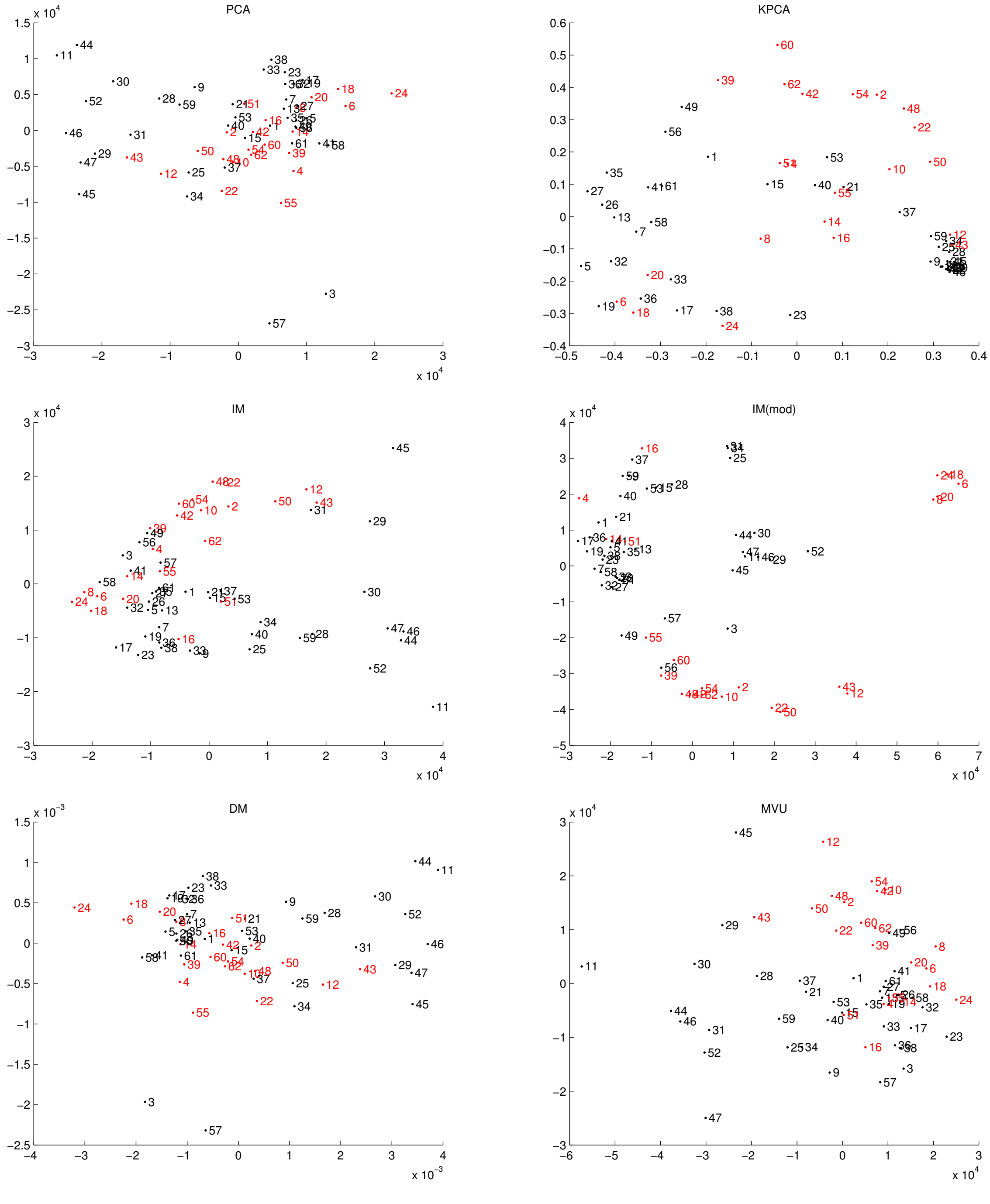

Figure S1: Visualization of the Alon et al. colon cancer dataset reduced to two dimensions by PCA and the global methods KPCA, IM, IM(mod), DM and MVU. Tumor samples were numbered in black, while normal samples are covered in red.

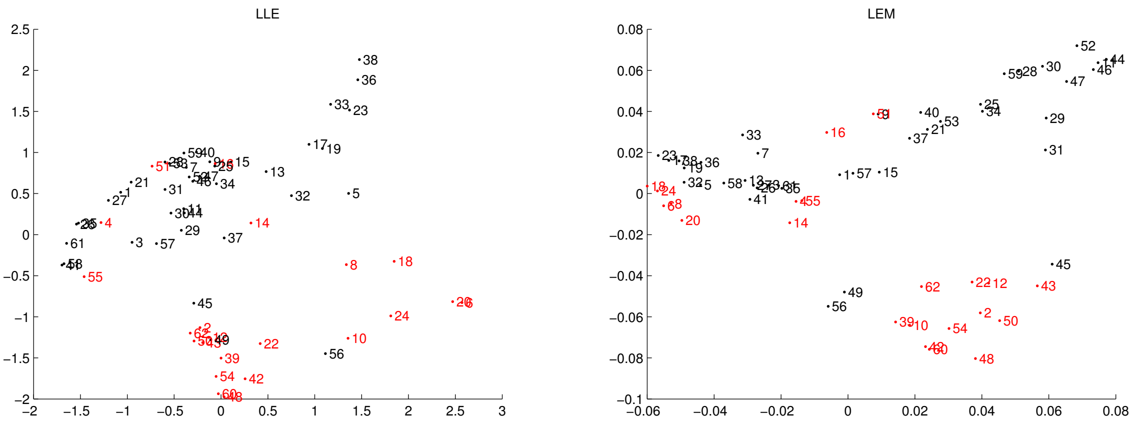

Figure S2: Visualization of the Alon et al. colon cancer dataset reduced to two dimensions by the local methods LLE and LEM. Tumor samples were numbered in black, while normal samples are covered in red.

Comparable to the cluster scores on page 12, PCA, Kernel PCA and DM show the least significant clusters. Both local methods, LLE and Laplacian Eigenmaps, separate best the tumor from the normal samples. Global methods like IM or MVU, however, might give a representation more true to the original, when preserving relations between all samples.

Except from PCA, Kernel PCA and DM, most methods share the same outliers, like the normal samples #16 and #51, lying in a neighbourhood of tumor samples.

4 Verhaak et al. - Leukemia dataset

method	dim	neighbours/σ	loo-cv accuracy
PCA	7	-	92.0
KPCA	8	100	92.4
LLE	14	16	91.5
IM	15	4	90.5
IM(mod)	15	12	88.5
LEM	10	6	88.2
DM	8	100	92.0
MVU	14	10	91.1

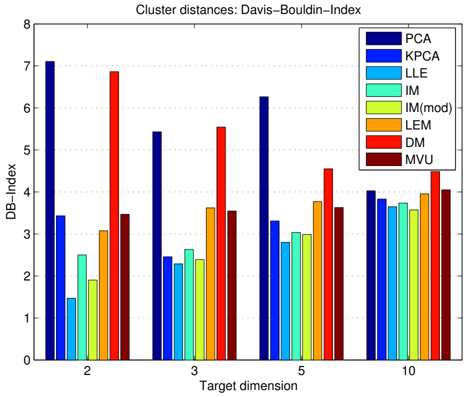

Figure S3: Left: All parameters of the dimension reduction techniques, estimated by leave-one-out cross-validation. PCA has no additional parameter, while KPCA and DM have a kernel parameter σ, the remaining methods take a number of neighbours as argument. Right: Davis-Bouldin-cluster-indices of the reduced data for fixed target space dimensions 2,3,5 and 10.

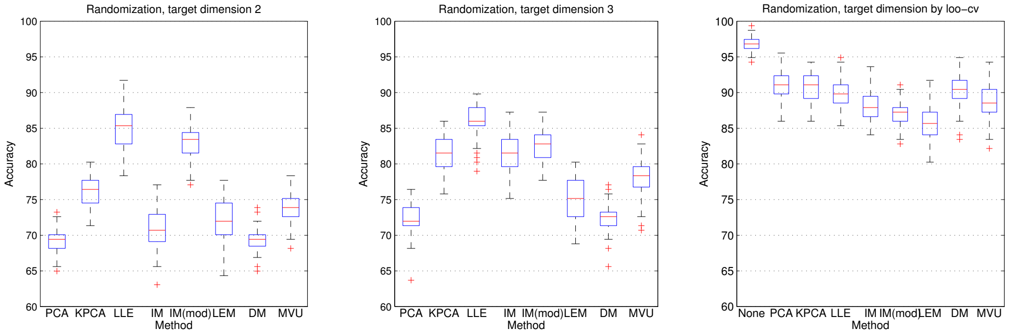

Figure S4: Support Vector Machine classification accuracies of the Verhaak et al. leukemia dataset. The data was randomized a hundred times for fixed target space dimensions two (left) and three (middle) and for dimensions estimated by loo-cv (right). In the last case, the plot also shows the results for the original high dimensional data without reduction.

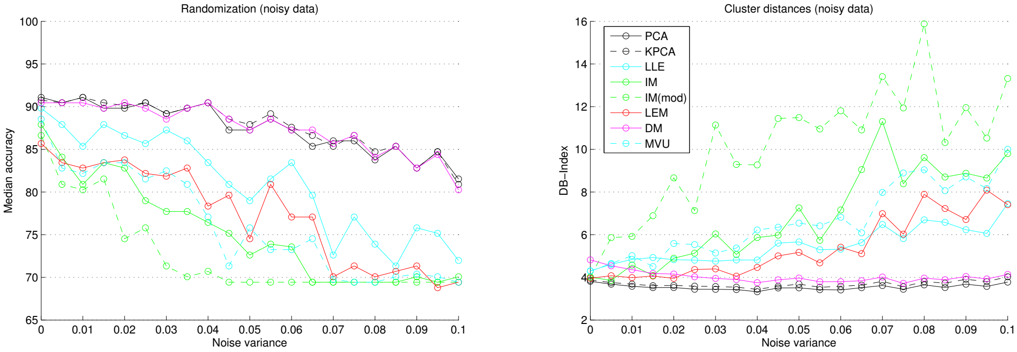

Figure S5: Randomization accuracies (left) and cluster-indices (right) for the Verhaak et al. leukemia dataset modified by normally distributed noise with zero mean and different variances.

5 Haferlach et al. - Leukemia dataset (NPM1 labeling)

method	dim	neighbours/σ	loo-cv accuracy
PCA	13	-	92.0
KPCA	13	1.000	92.0
LLE	13	14	87.3
IM	13	8	84.1
IM(mod)	15	6	81.7
LEM	7	4	79.7
DM	13	1.000	90.8
MVU	13	16	88.8

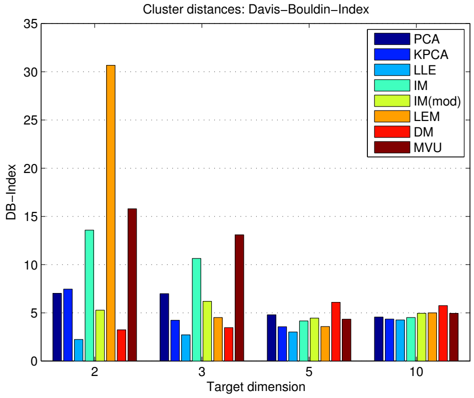

Figure S6: Left: All parameters of the dimension reduction techniques, estimated by leave-one-out cross-validation. PCA has no additional parameter, while KPCA and DM have a kernel parameter σ, the remaining methods take a number of neighbours as argument. Right: Davis-Bouldin-cluster-indices of the reduced data for fixed target space dimensions 2,3,5 and 10.

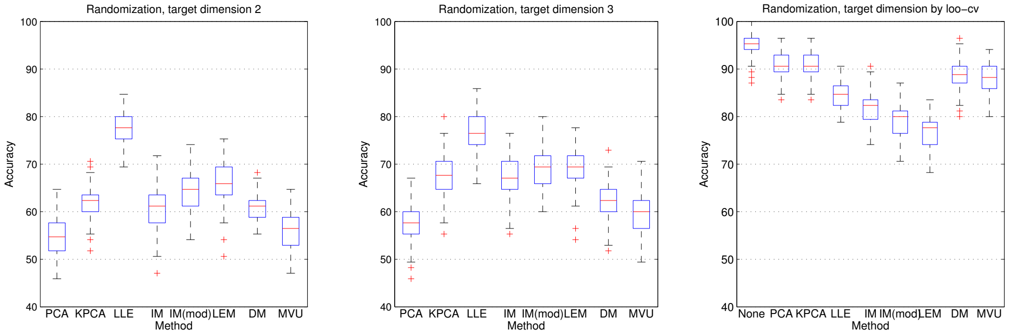

Figure S7: Support Vector Machine classification accuracies of the Haferlach et al. leukemia dataset. The data was randomized a hundred times for fixed target space dimensions two (left) and three (middle) and for dimensions estimated by loo-cv (right). In the last case, the plot also shows the results for the original high dimensional data without reduction.

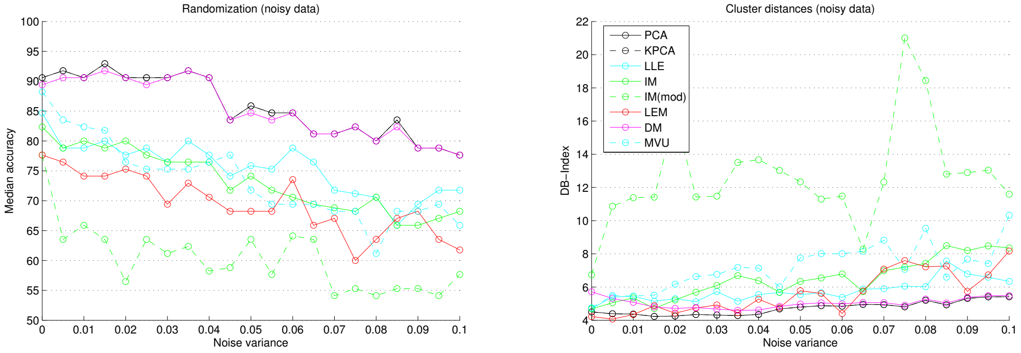

Figure S8: Randomization accuracies (left) and cluster-indices (right) for the Haferlach et al. leukemia dataset modified by normally distributed noise with zero mean and different variances.

6 Haferlach et al. - Leukemia dataset (AML labeling)

method	dim	neighbours/σ	loo-cv accuracy
PCA	3	-	96.1
KPCA	2	100	96.1
LLE	2	14	97.4
IM	3	12	98.7
IM(mod)	13	16	97.4
LEM	2	4	96.1
DM	3	1.000	96.1
MVU	3	14	97.4

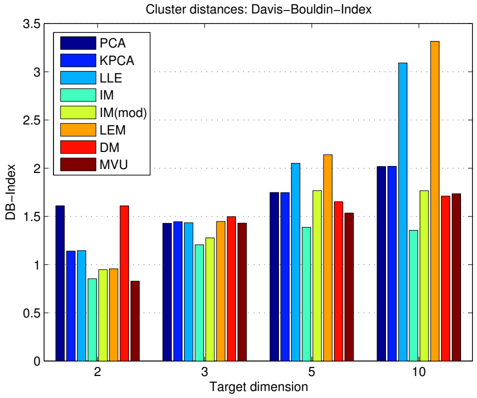

Figure S9: Left: All parameters of the dimension reduction techniques, estimated by leave-one-out cross-validation. PCA has no additional parameter, while KPCA and DM have a kernel parameter σ, the remaining methods take a number of neighbours as argument. Right: Davis-Bouldin-cluster-indices of the reduced data for fixed target space dimensions 2,3,5 and 10.

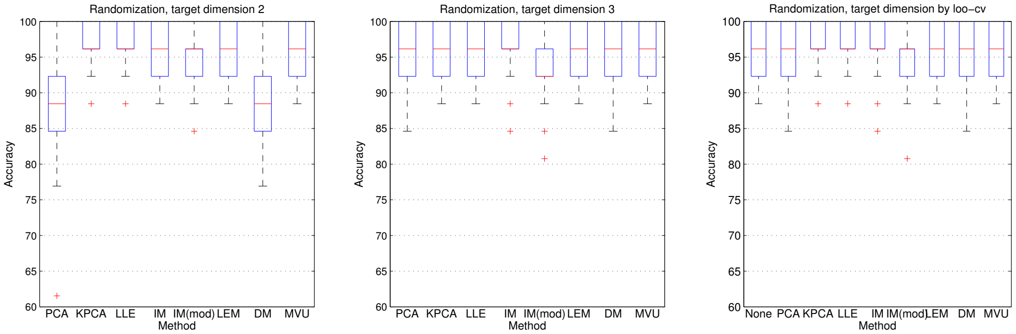

Figure S10: Support Vector Machine classification accuracies of the Haferlach et al. leukemia dataset. The data was randomized a hundred times for fixed target space dimensions two (left) and three (middle) and for dimensions estimated by loo-cv (right). In the last case, the plot also shows the results for the original high dimensional data without reduction.

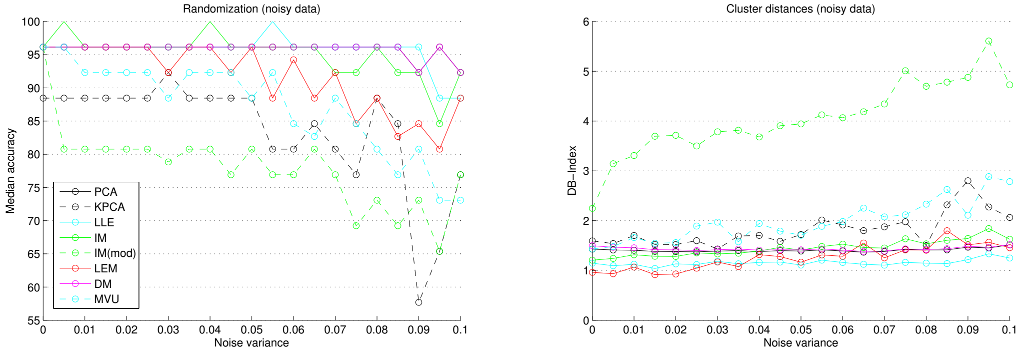

Figure S11: Randomization accuracies (left) and cluster-indices (right) for the Haferlach et al. leukemia dataset modified by normally distributed noise with zero mean and different variances.

7 Golub et al. - Leukemia dataset

method	dim	neighbours/σ	loo-cv accuracy
PCA	6	-	97.2
KPCA	4	1e5	97.2
LLE	10	8	98.6
IM	2	6	98.6
IM(mod)	2	12	93.1
LEM	8	4	97.2
DM	6	1e5	97.2
MVU	4	6	97.2

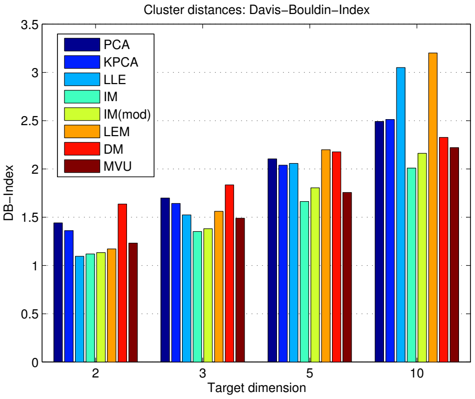

Figure S12: Left: All parameters of the dimension reduction techniques, estimated by leave-one-out cross-validation. PCA has no additional parameter, while KPCA and DM have a kernel parameter σ, the remaining methods take a number of neighbours as argument. Right: Davis-Bouldin-cluster-indices of the reduced data for fixed target space dimensions 2,3,5 and 10.

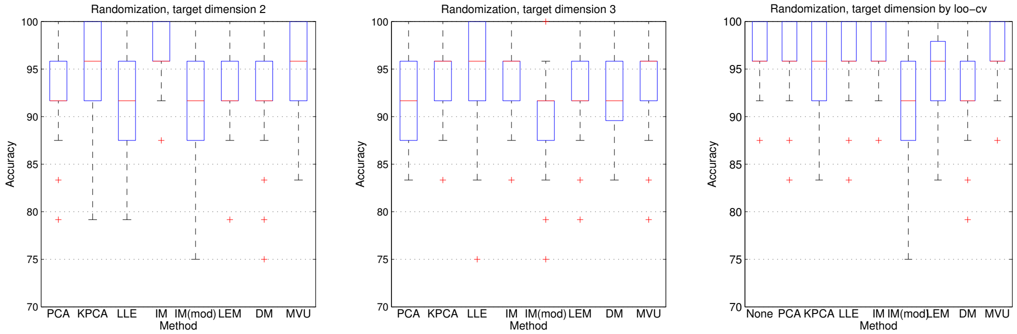

Figure S13: Support Vector Machine classification accuracies of the Golub et al. leukemia dataset. The data was randomized a hundred times for fixed target space dimensions two (left) and three (middle) and for dimensions estimated by loo-cv (right). In the last case, the plot also shows the results for the original high dimensional data without reduction.

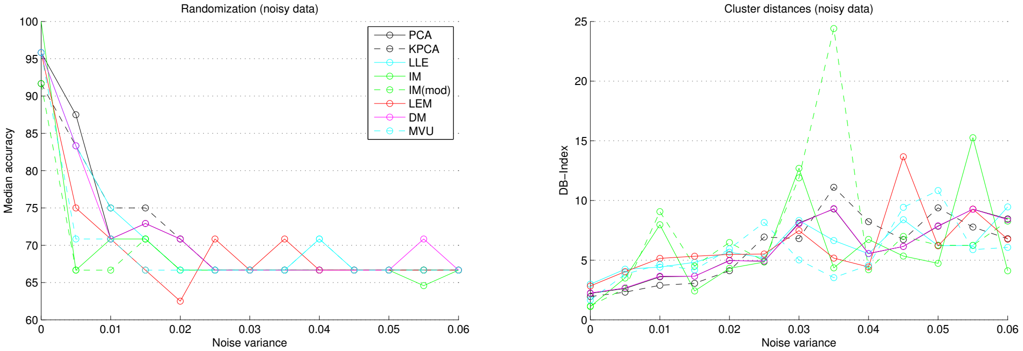

Figure S14: Randomization accuracies (left) and cluster-indices (right) for the Golub et al. leukemia dataset modified by normally distributed noise with zero mean and different variances.

8 Chiaretti et al. - Leukemia dataset

method	dim	neighbours/σ	loo-cv accuracy
PCA	13	-	90.9
KPCA	13	1.000	90.9
LLE	5	16	90.9
IM	3	6	95.5
IM(mod)	6	12	86.4
LEM	12	10	95.5
DM	4	100	86.4
MVU	3	14	90.9

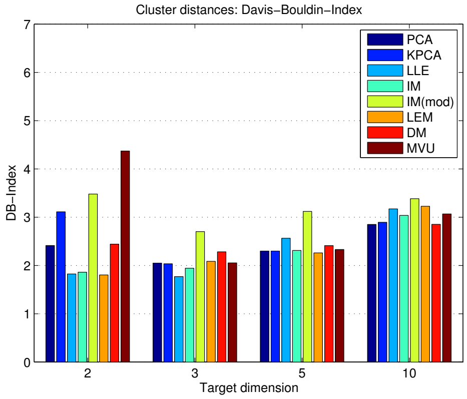

Figure S15: Left: All parameters of the dimension reduction techniques, estimated by leave-one-out cross-validation. PCA has no additional parameter, while KPCA and DM have a kernel parameter σ, the remaining methods take a number of neighbours as argument. Right: Davis-Bouldin-cluster-indices of the reduced data for fixed target space dimensions 2,3,5 and 10.

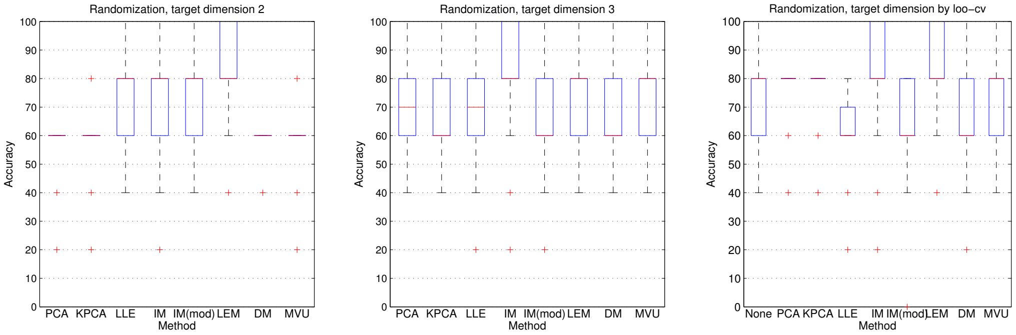

Figure S16: Support Vector Machine classification accuracies of the Chiaretti et al. leukemia dataset. The data was randomized a hundred times for fixed target space dimensions two (left) and three (middle) and for dimensions estimated by loo-cv (right). In the last case, the plot also shows the results for the original high dimensional data without reduction.

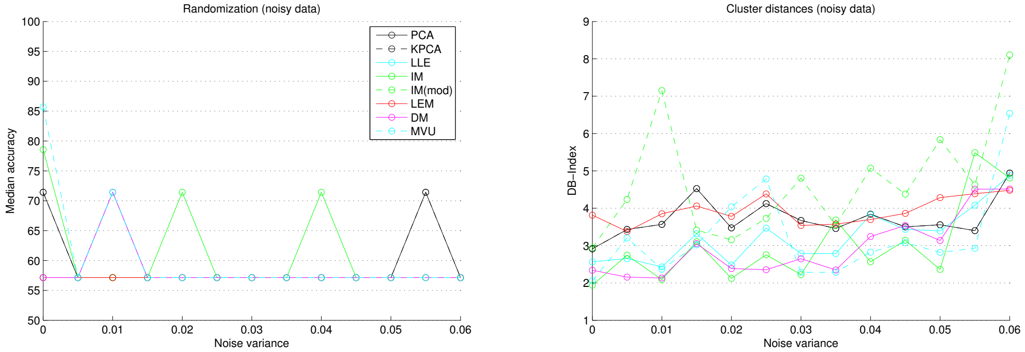

Figure S17: Randomization accuracies (left) and cluster-indices (right) for the Chiaretti et al. leukemia dataset modified by normally distributed noise with zero mean and different variances.

9 Alizadeh et al. - Lymphoma dataset

method	dim	neighbours/σ	loo-cv accuracy
PCA	8	-	94.7
KPCA	7	100	94.7
LLE	3	12	92.1
IM	7	6	100
IM(mod)	9	16	92.1
LEM	5	4	92.1
DM	15	1.000	97.4
MVU	6	16	94.7

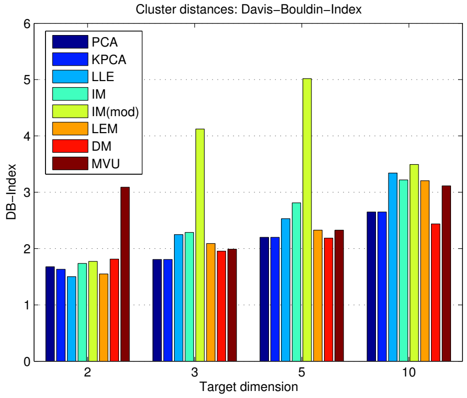

Figure S18: Left: All parameters of the dimension reduction techniques, estimated by leave-one-out cross-validation. PCA has no additional parameter, while KPCA and DM have a kernel parameter σ, the remaining methods take a number of neighbours as argument. Right: Davis-Bouldin-cluster-indices of the reduced data for fixed target space dimensions 2,3,5 and 10.

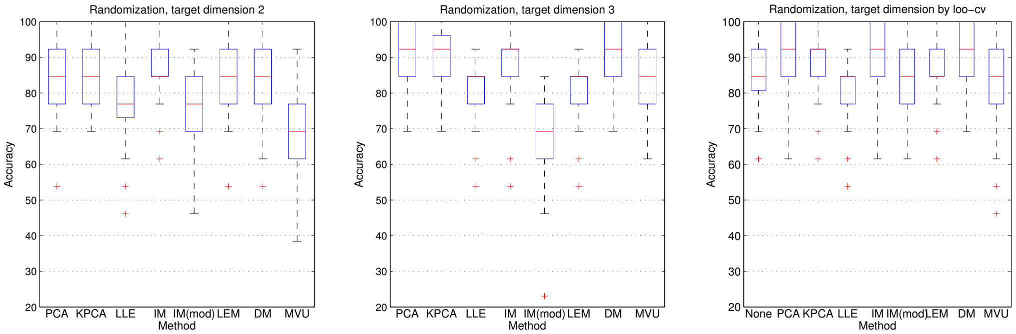

Figure S19: Support Vector Machine classification accuracies of the Alizadeh et al. lymphoma dataset. The data was randomized a hundred times for fixed target space dimensions two (left) and three (middle) and for dimensions estimated by loo-cv (right). In the last case, the plot also shows the results for the original high dimensional data without reduction.

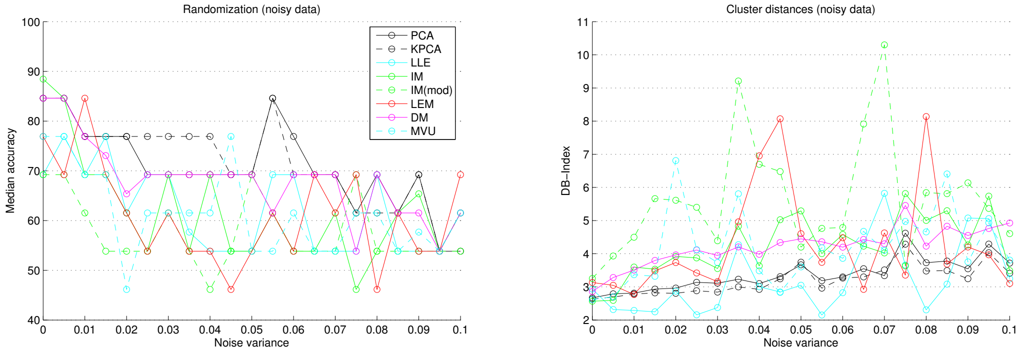

Figure S20: Randomization accuracies (left) and cluster-indices (right) for the Alizadeh et al. lymphoma dataset modified by normally distributed noise with zero mean and different variances.

10 Nutt et al. - High-grade glioma dataset

method	dim	neighbours/σ	loo-cv accuracy
PCA	4	-	84.0
KPCA	4	1.000	84.0
LLE	2	4	84.0
IM	7	6	86.0
IM(mod)	2	10	88.0
LEM	2	4	88.0
DM	8	50	86.0
MVU	3	4	90.0

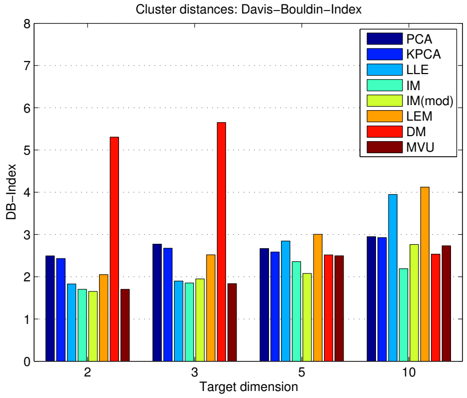

Figure S21: Left: All parameters of the dimension reduction techniques, estimated by leave-one-out cross-validation. PCA has no additional parameter, while KPCA and DM have a kernel parameter σ, the remaining methods take a number of neighbours as argument. Right: Davis-Bouldin-cluster-indices of the reduced data for fixed target space dimensions 2,3,5 and 10.

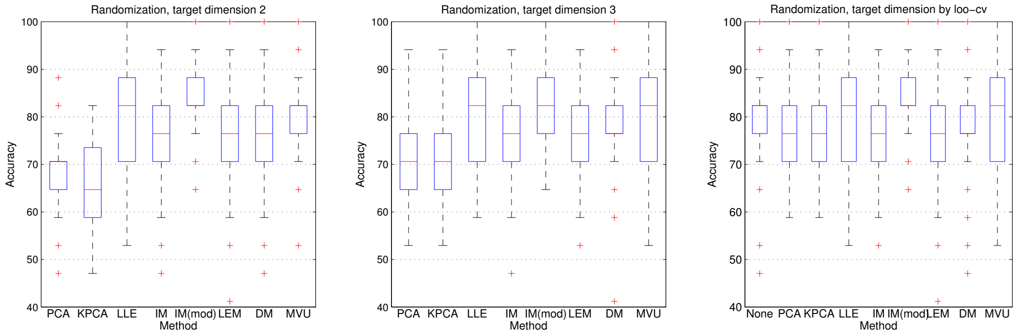

Figure S22: Support Vector Machine classification accuracies of the Nutt et al. high-grade glioma dataset. The data was randomized a hundred times for fixed target space dimensions two (left) and three (middle) and for dimensions estimated by loo-cv (right). In the last case, the plot also shows the results for the original high dimensional data without reduction.

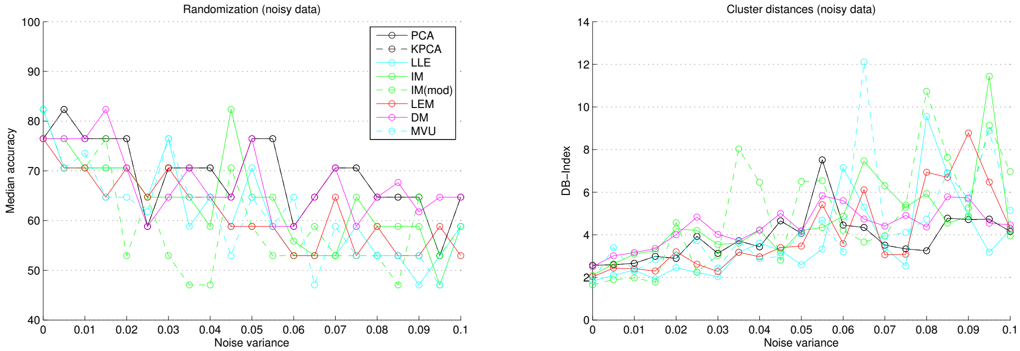

Figure S23: Randomization accuracies (left) and cluster-indices (right) for the Nutt et al. high-grade glioma dataset modified by normally distributed noise with zero mean and different variances.

11 Alon et al. - Colon cancer dataset

method	dim	neighbours/σ	loo-cv accuracy
PCA	10	-	90.3
KPCA	3	1e4	90.3
LLE	7	4	88.7
IM	6	8	88.7
IM(mod)	2	4	90.3
LEM	2	8	91.9
DM	7	5e4	88.7
MVU	8	12	90.3

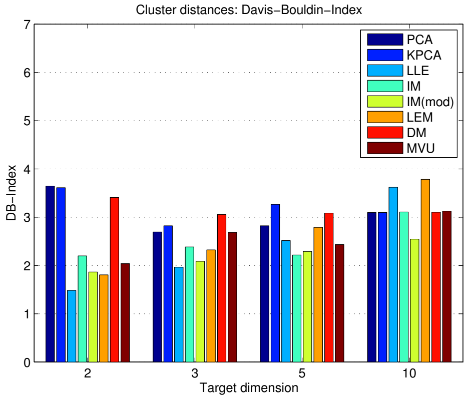

Figure S24: Left: All parameters of the dimension reduction techniques, estimated by leave-one-out cross-validation. PCA has no additional parameter, while KPCA and DM have a kernel parameter σ, the remaining methods take a number of neighbours as argument. Right: Davis-Bouldin-cluster-indices of the reduced data for fixed target space dimensions 2,3,5 and 10.

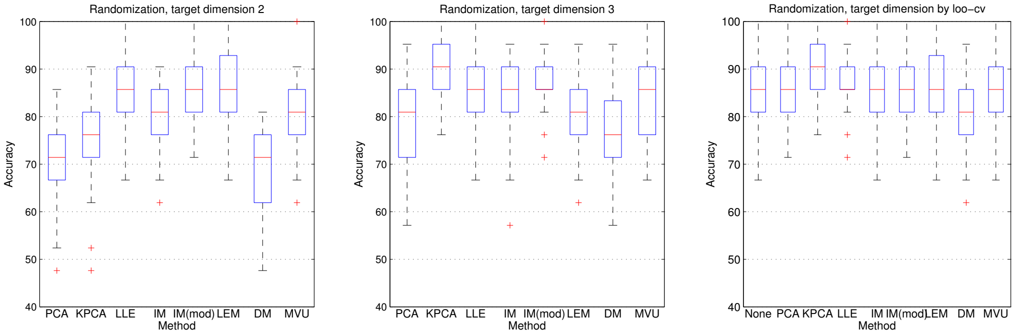

Figure S25: Support Vector Machine classification accuracies of the Alon et al. colon cancer dataset. The data was randomized a hundred times for fixed target space dimensions two (left) and three (middle) and for dimensions estimated by loo-cv (right). In the last case, the plot also shows the results for the original high dimensional data without reduction.

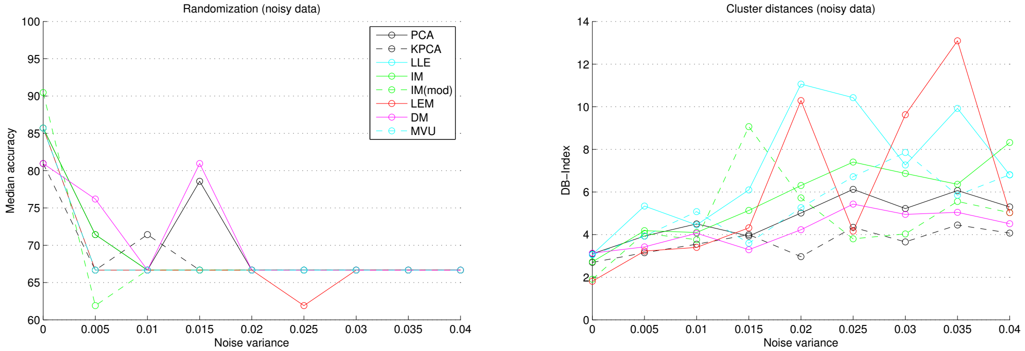

Figure S26: Randomization accuracies (left) and cluster-indices (right) for the Alon et al. colon cancer dataset modified by normally distributed noise with zero mean and different variances.

12 Singh et al. - Prostate cancer dataset

method	dim	neighbours/σ	loo-cv accuracy
PCA	9	-	92.2
KPCA	9	1.000	92.2
LLE	15	14	89.2
IM	6	10	86.3
IM(mod)	5	6	86.3
LEM	15	8	85.3
DM	14	1.000	90.2
MVU	5	10	93.1

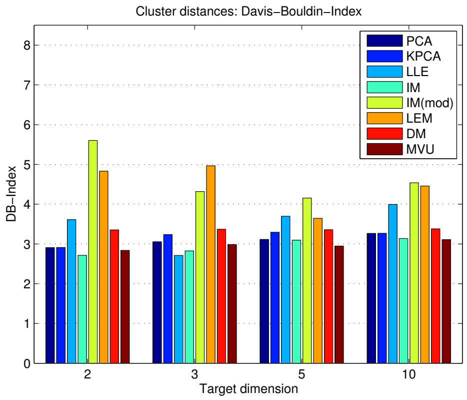

Figure S27: Left: All parameters of the dimension reduction techniques, estimated by leave-one-out cross-validation. PCA has no additional parameter, while KPCA and DM have a kernel parameter σ, the remaining methods take a number of neighbours as argument. Right: Davis-Bouldin-cluster-indices of the reduced data for fixed target space dimensions 2,3,5 and 10.

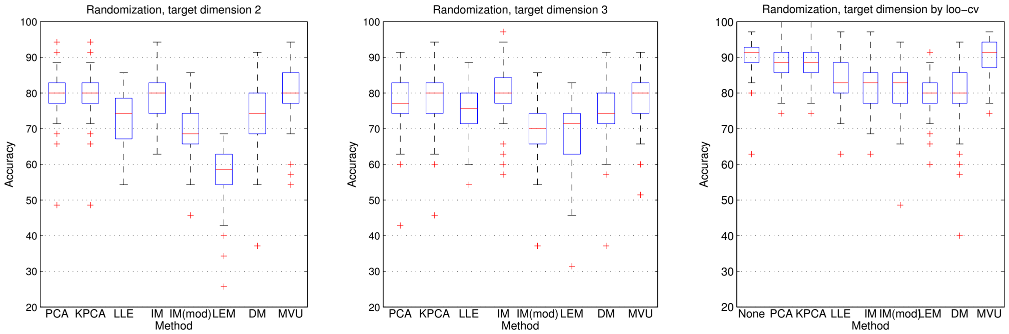

Figure S28: Support Vector Machine classification accuracies of the Singh et al. prostate cancer dataset. The data was randomized a hundred times for fixed target space dimensions two (left) and three (middle) and for dimensions estimated by loo-cv (right). In the last case, the plot also shows the results for the original high dimensional data without reduction.

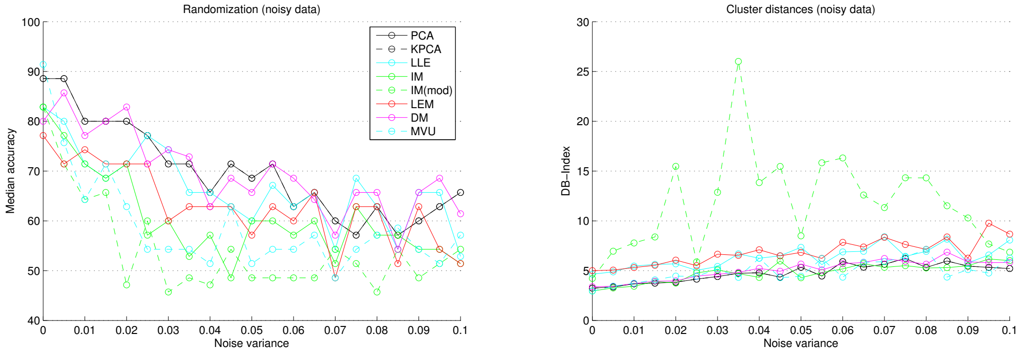

Figure S29: Randomization accuracies (left) and cluster-indices (right) for the Singh et al. prostate cancer dataset modified by normally distributed noise with zero mean and different variances.

13 Simulated data - Randomization for three target dimensions

The original article already discussed the randomization results for the simulated datasets. Figure S30 below continues the benchmark for three target dimensions.

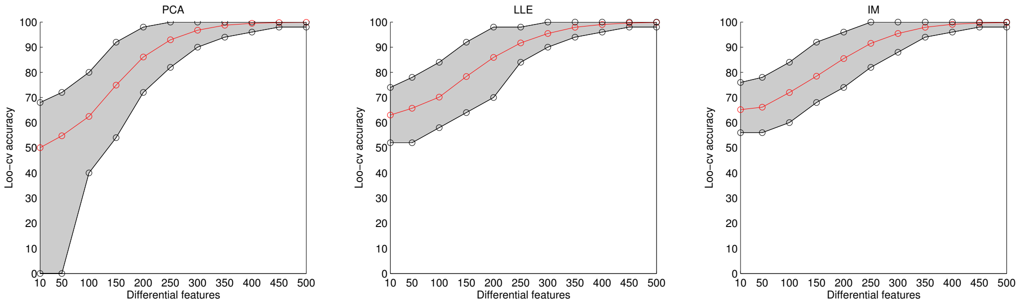

Figure S30: Leave-one-out cross-validation accuracies of the simulated datasets with increasing differential features. The red line marks the average accuracy of all 100 generated datasets within the 95% quantile. In three dimensional target spaces, LLE an IM capture more of the underlying structure of the data with much fewer significant features than PCA.

14 Simulated data - Noise evaluation

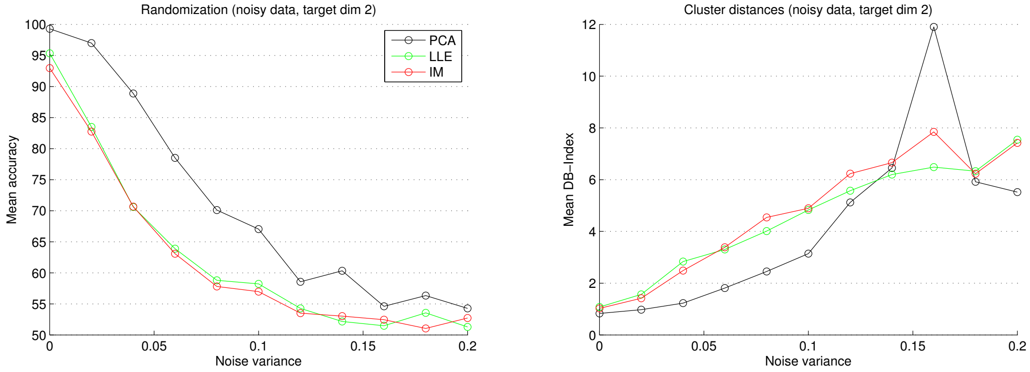

Figure S31: Mean randomization accuracies (left) and cluster-indices (right) of 50 simulated datasets combined with normally distributed noise with zero mean and variances between 0 and 0.2. Target dimension for PCA, LLE and Isomap was set to 2.

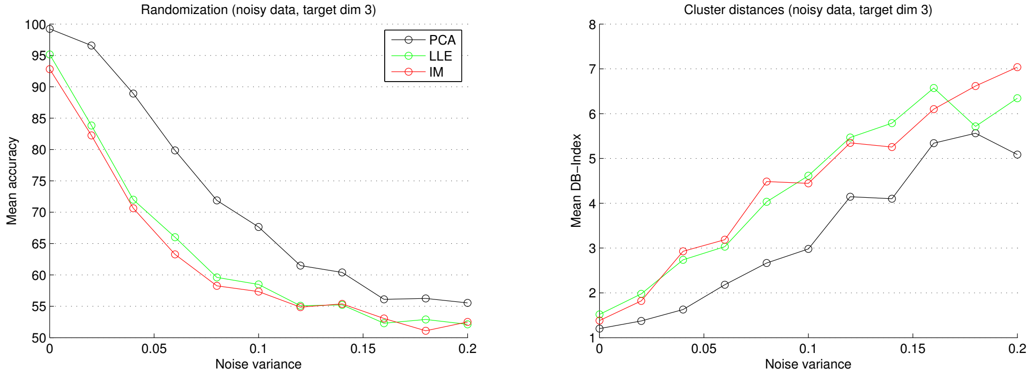

Figure S32: Mean randomization accuracies (left) and cluster-indices (right) of 50 simulated datasets combined with normally distributed noise with zero mean and variances between 0 and 0.2. Target dimension for PCA, LLE and Isomap was set to 3.

References

1. Wang Y, Klijn JG, Zhang Y, Sieuwerts AM, Look MP, Yang F, Talantov D, Timmermans M, van Gelder MEM, Yu J, Jatkoe T, Berns EM, Atkins D, Foekens JA: **Gene-expression profiles to predict distant metastasis of lymph-node-negative primary breast cancer**. *The Lancet* 2005, **365**(9460):671 – 679.

2. Verhaak R, Wouters B, Erpelinck C, Abbas S, Beverloo H, Lugthart S, Löwenberg B, Delwel R, Valk P: **Prediction of molecular subtypes in acute myeloid leukemia based on gene expression profiling**. *Haematologica* 2009, **94**:131 – 134.

3. Klein HU, Ruckert C, Kohlmann A, Bullinger L, Thiede C, Haferlach T, Dugas M: **Quantitative comparison of microarray experiments with published leukemia related gene expression signatures**. *BMC Bioinformatics* 2009, **10**:422.

4. Golub TR, Slonim DK, Tamayo P, Huard C, Gaasenbeek M, Mesirov JP, Coller H, Loh ML, Downing JR, Caligiuri MA, Bloomfield CD: **Molecular classification of cancer: class discovery and class prediction by gene expression monitoring**. *Science* 1999, **286**:531–537.

5. Del Giudice I, Chiaretti S, Tavolaro S, De Propris MS, Maggio R, Mancini F, Peragine N, Santangelo S, Marinelli M, Mauro FR, Guarini A, Foa R: **Spontaneous regression of chronic lymphocytic leukemia: clinical and biologic features of 9 cases**. *Blood* 2009, **114**(3):638–646.

6. Powell JI, Yang L, Marti GE, Moore T, Hudson J, Lu L, Lewis DB, Tibshirani R, Sherlock G, Chan ea W C: **Distinct types of diffuse large B-cell lymphoma identified by gene expression profiling**. *Nature* 2000, **403**(6769):503–511.

7. Nutt CL, Mani DR, Betensky RA, Tamayo P, Cairncross JG, Ladd C, Pohl U, Hartmann C, McLaughlin ME, Batchelor TT, Black PM, von Deimling A, Pomeroy SL, Golub TR, Louis DN: **Gene expression-based classification of malignant gliomas correlates better with survival than histological classification**. *Cancer Research* 2003, **63**(7):1602–1607.

8. Alon U, Barkai N, Notterman DA, Gish K, Ybarra S, Mack D, Levine AJ: **Broad patterns of gene expression revealed by clustering analysis of tumor and normal colon tissues probed by oligonucleotide arrays**. *Proceedings of the National Academy of Sciences of the United States of America* 1999, **96**(12):6745–6750.

9. Singh D, Febbo PG, Ross K, Jackson DG, Manola J, Ladd C, Tamayo P, Renshaw AA, D'Amico AV, Richie JP, Lander ES, Loda M, Kantoff PW, Golub TR, Sellers WR: **Gene expression correlates of clinical prostate cancer behavior**. *Cancer Cell* 2002, **1**(2):203 – 209.

10. **Lymphoma/Leukemia Molecular Profiling Project.** [`http://llmpp.nih.gov/lymphoma/`, last accessed at 29th of Oct 2010].

11. **Gene expression-based classification of malignant gliomas correlates better with survival than histological classification.** [`http://www.broadinstitute.org/cgi-bin/cancer/publications/pub_paper.cgi?mode=view&paper_id=82`, last accessed at 29th of Oct 2010].

12. **Data pertaining to the article Broad patterns of gene expression revealed by clustering of tumor and normal colon tissues probed by oligonucleotide arrays.** [`http://genomics-pubs.princeton.edu/oncology/affydata/index.html`, last accessed at 29th of Oct 2010].

13. **Gene Expression Correlates of Clinical Prostate Cancer Behavior.** [`http://www.broadinstitute.org/cgi-bin/cancer/publications/pub_paper.cgi?mode=view&paper_id=75`, last accessed at 29th of Oct 2010].
